# Nanocrystal Heterostructures Based on Halide Perovskites and Metal Sulfides

**DOI:** 10.1021/jacs.4c08565

**Published:** 2024-09-30

**Authors:** Nikolaos Livakas, Juliette Zito, Yurii P. Ivanov, Clara Otero-Martínez, Giorgio Divitini, Ivan Infante, Liberato Manna

**Affiliations:** † Nanochemistry, Istituto Italiano di Tecnologia, Via Morego 30, Genova 16163, Italy; ‡ Dipartimento di Chimica e Chimica Industriale, Università di Genova, Genova 16146, Italy; § Electron Spectroscopy and Nanoscopy, Istituto Italiano di Tecnologia, Via Morego 30, Genova 16163, Italy; ∥ Department of Physical Chemistry, Materials Chemistry and Physics Group, Universidade de Vigo, Campus Universitario As Lagoas-Marcosende, CINBIO, Vigo 36310, Spain; ⊥ UPV/EHU Science Park, BCMaterials, Basque Center for Materials, Applications, and Nanostructures, Leioa 48940, Spain; # Ikerbasque Basque Foundation for Science, Bilbao 48009, Spain

## Abstract

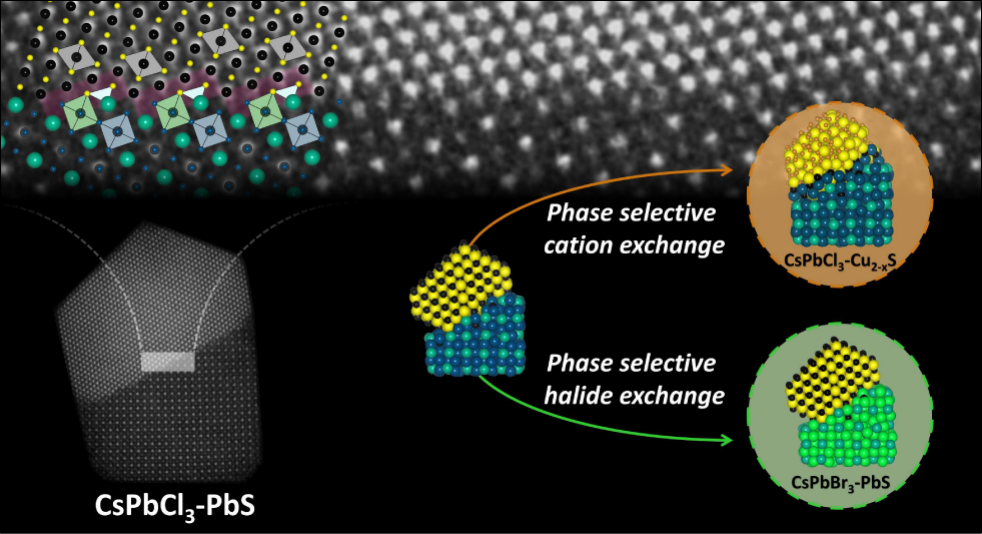

We report the synthesis of nanocrystal heterostructures composed of CsPbCl_3_ and PbS domains sharing an epitaxial interface. We were able to promote the growth of a PbS domain (in competition with the more commonly observed Pb_4_S_3_Cl_2_ one) on top of the CsPbCl_3_ domain by employing Mn^2+^ ions, the latter most likely acting as scavengers of Cl^–^ ions. Complete suppression of the Pb_4_S_3_Cl_2_ domain growth was then achieved by additionally selecting an appropriate sulfur source (bis(trimethylsilyl)sulfide, which also acted as a scavenger of Cl^–^ ions) and reaction temperature. In the heterostructures, emission from the perovskite domain was quenched, while emission from the PbS domain was observed, pointing to a type-I band alignment, as confirmed by calculations. These heterostructures, in turn, could be exploited to prepare second-generation heterostructures through selective ion exchange on the individual domains (halide ion exchange on CsPbCl_3_ and cation exchange on PbS). We demonstrate the cases of Cl^–^ → Br^–^ and Pb^2+^ → Cu^+^ exchanges, which deliver CsPbBr_3_–PbS and CsPbCl_3_–Cu_2-*x*_S epitaxial heterostructures, respectively.

## Introduction

Colloidal semiconductor nanocrystals (NCs) of all-inorganic lead halide perovskites (LHPs) (CsPbX_3_, X = Cl^–^, Br^–^, I^–^) have attracted broad research interest owing to their notable optoelectronic properties, such as high absorption coefficients and photoluminescence quantum yields (PLQYs), narrow emission bandwidth, and defect tolerance.^1−5^ More recently, research has also expanded in the direction of colloidal NC heterostructures, in which one domain is a halide perovskite.^6−23^ Traditionally, heterostructures with heterodimer or core–shell geometries have been proven beneficial in terms of stability,^24^ bandgap tunability,^25,26^ and the emergence of new properties,^27^ leading to their extensive investigation in light-emitting devices,^28,29^ catalysis,^28^ and biomedicine.^30^ For instance, in a core/shell geometry with a type-I band alignment, the shell generally improves the stability and PLQY of the light emitted from the core,^31^ whereas in a heterodimer geometry in which the two domains have a type-II band alignment, opposite charge carriers can be separated across the heterojunction and can then be individually exploited for catalytic applications.^24,32^

To date, various materials have been coupled to LHPs to form heterostructures or core/shell structures. The most studied cases are metal oxides (SiO_2_ and TiO_2_),^33−36^ metals (Au, Pt, and Bi),^17−20^ metal halides (Cs_4_PbBr_6_, CsBr),^11,15,16^ chalcogenides (CdS, PbS),^10,11,13,14,37−39^ dichalcogenides (MoSe_2_),^40^ and chalcohalides (Pb_4_S_3_Br_2_).^9,12,23^ Only in a subset of these works was the interface between the LHP and the second material studied in detail and found to be epitaxial. A case study of an epitaxial interface was reported by our group, involving CsPbBr_3_ and the chalcohalide Pb_4_S_3_Br_2_.^23^ In it, the emission from the LHP domain was quenched by the presence of the chalcohalide domain. In a later work, those heterostructures were found to promote photocatalytic CO_2_ reduction.^12^ Research in this direction was extended by us to the CsPbCl_3_–Pb_4_S_3_Cl_2_ case.^9^ Another recent example of heteroepitaxy is the one between CsPbBr_3_ and CdS, a type-II heterojunction providing improved sensitivity in photodetection.^11^ An example of a type-I alignment, and the focus of this work, is found in CsPbBr_3_–PbS. Although in previous works there have been attempts to grow this type of heterostructure and study its properties, no clear evidence was presented of an epitaxial connection between the two domains.^13,39^

**Scheme 1:**
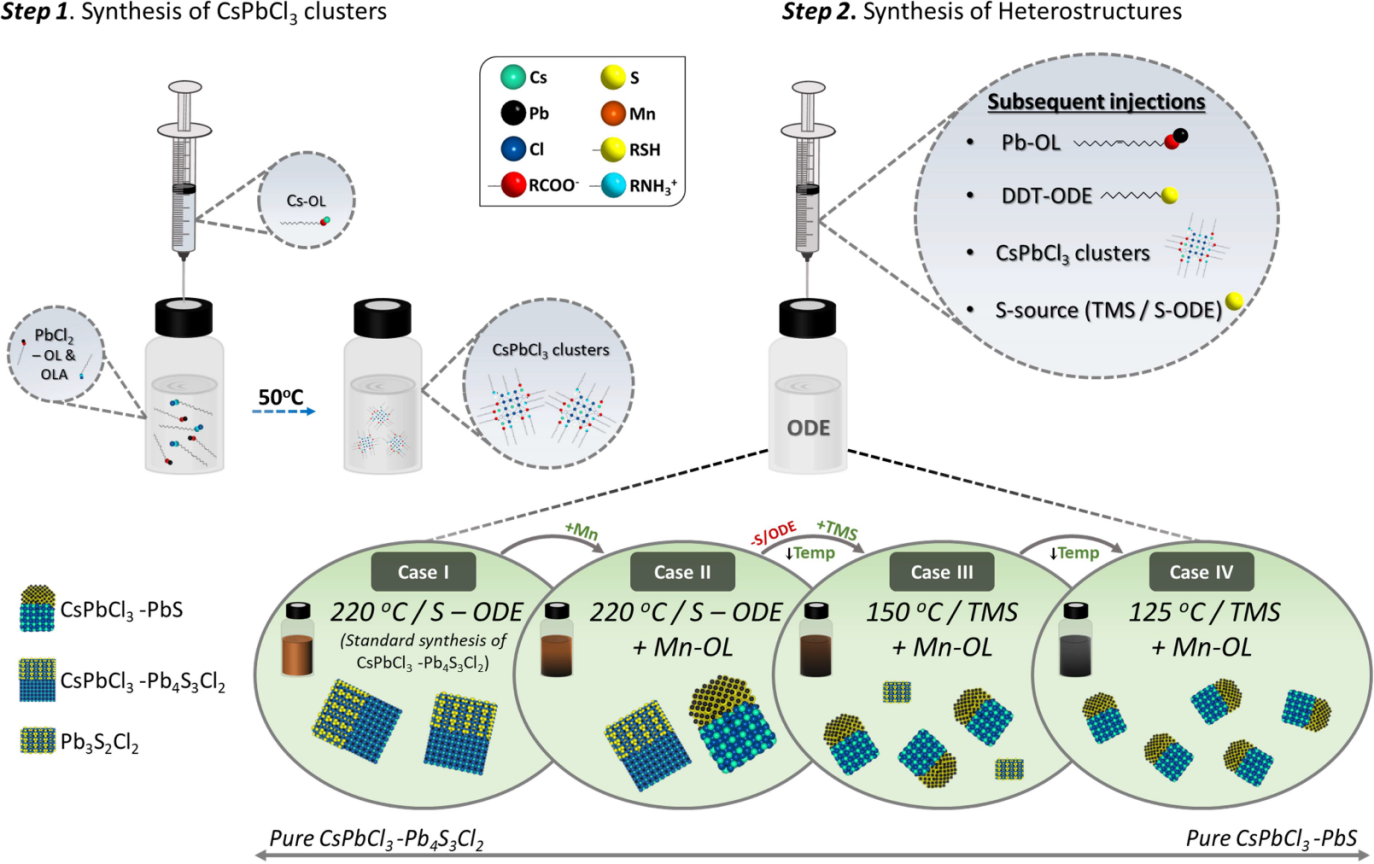
Heterostructure Synthesis: Step 1: Synthesis of CsPbCl_3_ Nanoclusters; Step 2: Synthesis of Heterostructures by Mixing the CsPbCl_3_ Nanoclusters with Different Reactants under Various Reaction Conditions Case I: our previously published protocol, leading to CsPbCl_3_–Pb_4_S_3_Cl_2_ heterostructures.^9^ Case II: including Mn-oleate promotes the growth of CsPbCl_3_–PbS in addition to CsPbCl_3_–Pb_4_S_3_Cl_2_. Case III: replacing S-ODE with a more reactive sulfur source (TMS) and working at a lower temperature (150 °C) leads to CsPbCl_3_–PbS as the only heterostructures, albeit smaller than in Case II, and with isolated chalcohalides as a byproduct. Case IV: CsPbCl_3_–PbS heterostructures are obtained as the only product at even lower temperatures (125 °C), with an overall size smaller than in Case III.

We report here a synthesis route for CsPbCl_3_–PbS epitaxial NC heterostructures. Our approach is similar to the one previously developed by us to grow CsPbCl_3_–Pb_4_S_3_Cl_2_ heterostructures:^9^ in that case, preformed CsPbCl_3_ clusters (Scheme 1, “synthesis of CsPbCl_3_ clusters”) were injected into a reaction environment that promotes their evolution to larger CsPbCl_3_ NCs, followed by the heterogeneous nucleation of a Pb_4_S_3_Cl_2_ domain on them (Scheme 1, “synthesis of heterostructures”, Case I). This latter domain grows using a fraction of Pb^2+^ and Cl^–^ ions derived from the decomposition of the initial CsPbCl_3_ clusters plus a sulfur source. In the present work, we realized that to selectively promote the heterogeneous nucleation of PbS over that of Pb_4_S_3_Cl_2_ (thus forming CsPbCl_3_–PbS heterostructures instead of CsPbCl_3_–Pb_4_S_3_Cl_2_ ones), we had to reduce the availability of the Cl^–^ species in solution. This was made possible by the addition to the reaction environment of an exogenous cation, Mn^2+^, which does not participate in the crystallization processes, yet, likely by binding to Cl^–^ ions in the solution phase, reduces their availability and thus promotes the heterogeneous nucleation of PbS instead of Pb_4_S_3_Cl_2_ (Scheme 1, Cases II–IV).

We found that there is strong competition between the chalcogenide (PbS) and chalcohalide (Pb_4_S_3_Cl_2_) phases to heterogeneously nucleate on the CsPbCl_3_ domain to the point that there is a narrow window of experimental parameters in which PbS prevails. The growth of CsPbCl_3_–PbS heterostructures carrying CsPbCl_3_ and PbS domains that are large enough (15 nm or bigger) for an accurate characterization of the epitaxial interface by transmission electron microscopy (TEM) comes at the cost of a conspicuous copresence of CsPbCl_3_–Pb_4_S_3_Cl_2_ heterostructures (Scheme 1, Case II). Instead, purer, although smaller-sized CsPbCl_3_–PbS heterostructures were grown using a more reactive sulfur precursor (bis(trimethylsilyl)sulfide), which could also scavenge Cl^–^ ions through its reactive trimethylsilyl groups, and working at comparatively lower temperatures (125–150 °C instead of 220 °C, Scheme 1, Cases III and IV). Scanning transmission electron microscopy (STEM) analysis indicated a well-defined epitaxial interface between the perovskite and lead sulfide domains due to the continuity of the Pb sublattice across the interface between the two domains: they share a common Pb layer at the junction, which coordinates with Cl^–^ ions on one side and S^2–^ ions on the other side. Density functional theory (DFT) calculations suggested a quasi-type-I band alignment at the heterojunction, with band-edge electrons fully localized in the PbS domain and holes that are instead localized in a region at the interface of the two domains, spanning both of them. These calculations are in accordance with the optical emission spectra of the heterostructures exhibiting PL in the near-infrared (NIR) region (originating from PbS), but weak PL in the blue region (originating from CsPbCl_3_), indicative of predominant carrier transfer from CsPbCl_3_ to the PbS domain upon photoexcitation.

Our samples were then used as precursors for the synthesis of second-generation heterostructures by exploiting the selectivity of the two domains to different ion exchange reactions. For example, by exchanging Pb^2+^ ions with Cu^+^ ions, we could transform the PbS domain to Cu_2-*x*_S, delivering CsPbCl_3_–Cu_2-*x*_S heterostructures. Alternatively, by exchanging Cl^–^ ions with Br^–^ ions, we could transform the CsPbCl_3_ domain to CsPbBr_3_, delivering CsPbBr_3_–PbS heterostructures. In both cases, the epitaxial connection between the ion-exchanged and the unexchanged domains was maintained. Our approach demonstrates a flexible strategy to synthesize NC heterostructures in which one of the two domains is a halide perovskite and the other is a metal chalcogenide.

## Results and Discussion

The synthesis of CsPbCl_3_–PbS colloidal heterostructure NCs was carried out via the cluster-based approach (Scheme 1). This is a two-step method that has been established as an effective route for the synthesis of several perovskite-based heterostructures.^9,10,23^ The first step in this method includes the synthesis of CsPbCl_3_ nanoclusters at a relatively low temperature (50 °C) over a rather long reaction time (30 min), made possible by a high concentration of surfactants, namely oleic acid and oleylamine (see details in the Experimental Section).^41^ These clusters are then isolated, purified, and resuspended in octadecene (ODE), which are used as precursors in the second step. This latter step is a modification of our previously developed synthesis of CsPbCl_3_–Pb_4_S_3_Cl_2_ heterostructures and consists of sequential injections, into degassed ODE at high temperature (220 °C), of Pb-oleate, dodecanethiol, preformed CsPbCl_3_ nanoclusters, and elemental sulfur (dissolved in ODE, henceforth referred to as S-ODE).^23^

**Figure 1:**
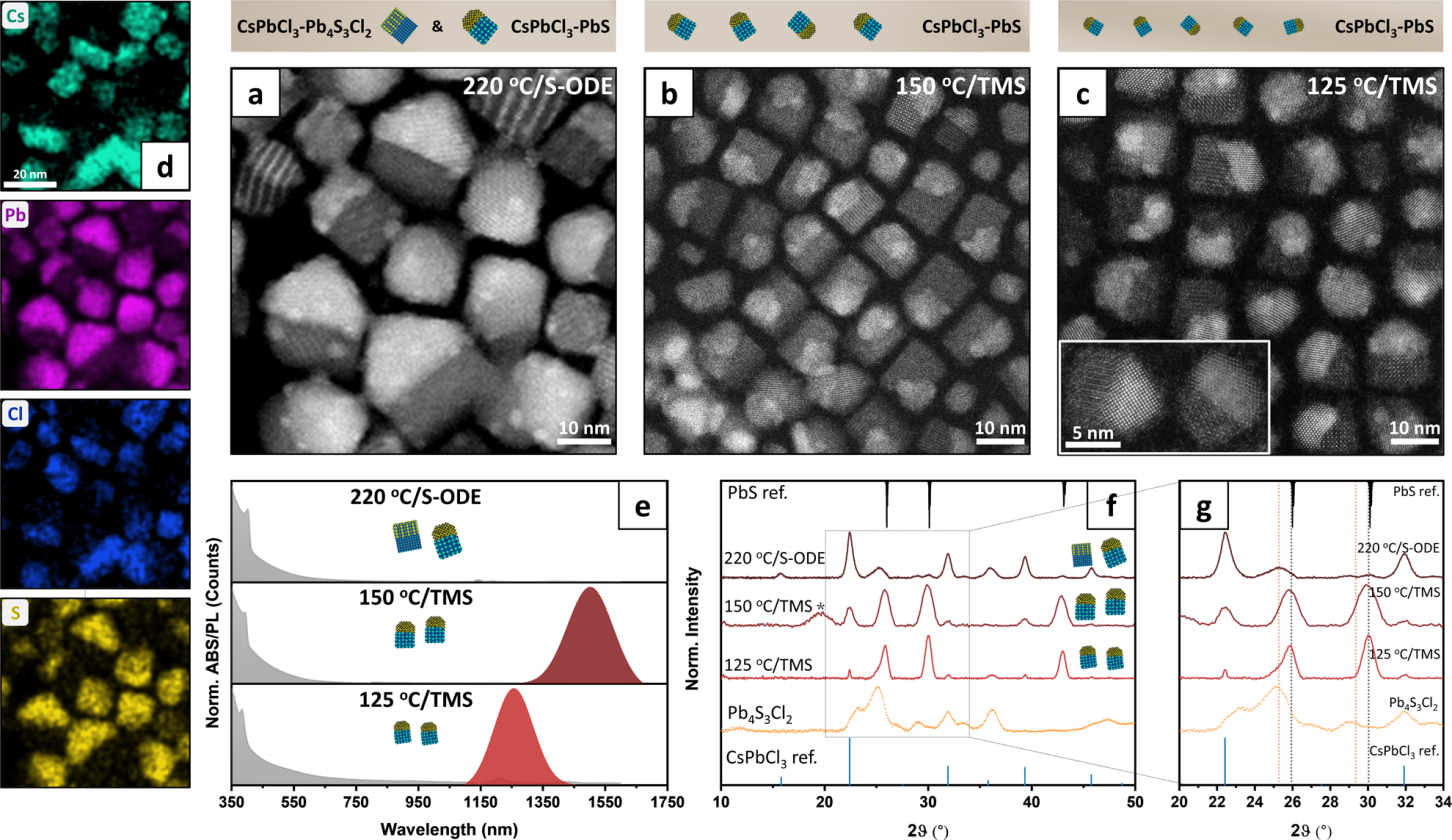
Analysis of heterostructures from Cases II, III, and IV of Scheme 1. (a–c) HAADF-STEM images of each sample. (d) STEM-EDX elemental maps of the same area represented in (a) demonstrate the coexistence of two types of heterostructures in this sample. (e) Optical absorption (gray) and PL (colored) spectra of the three samples (the “220 °C/S-ODE” sample does not have any NIR emission). (f) XRD patterns of the three samples and Pb_4_S_3_Cl_2_, highlighting the CsPbCl_3_–PbS phase purity for the “125 °C/TMS” and “150 °C/TMS” samples, and the coexistence of the two types of heterostructures in the “220 °C/S-ODE” sample. The peak at 20° (marked with *), is attributed to fumed silica used to prepare XRD samples. (g) Magnified view of the same XRD patterns in the 20°–34° region, highlighting the presence of the pure PbS phase in the “125 °C/TMS” and “150 °C/TMS” samples, with distinctive diffraction peaks at 26° and 30° (black dashed lines). The “220 °C/S-ODE” sample presents a dominant chalcohalide-based composition, as indicated by the diffraction peaks at 25.2° and 29.1° (orange dashed lines).

In this work, the only additional ingredient is represented by Mn^2+^ ions, introduced as Mn-oleate. Initially, the feed ratio *X*_Mn_ of Mn (defined as *X*_Mn_ = [Mn]/([Mn] + [Pb]) × 100) was 33%. This synthesis led to a mixture of the usual CsPbCl_3_/–Pb_4_S_3_Cl_2_ heterostructures along with larger, mushroom-like heterostructures (Figure 1a), which were identified as CsPbCl_3_–PbS based on high-resolution scanning TEM energy dispersive X-ray spectroscopy (HR STEM-EDX, Figure 1d). The coexistence of these two populations of heterostructures was further confirmed through X-ray diffraction (XRD, Figure 1f,g). The presence of Mn^2+^ was instrumental in the competitive formation of CsPbCl_3_–PbS, but only trace amounts of Mn were actually found in the NCs, mainly in the PbS domain (see STEM-EDX elemental mapping in Figure S1 and the corresponding analysis in Table S1). We therefore concluded that Mn^2+^ ions influence the kinetics of heterogeneous nucleation of PbS and Pb_4_S_3_Cl_2_, most likely by complexing a fraction of the available Cl^–^ ions in solution. This is supported by the stronger bond dissociation energy of Mn–Cl (361 kJ/mol) compared to Pb–Cl (301 kJ/mol),^42^ and our DFT calculations of the binding affinity of Cl^–^ ions to Pb^2+^ and Mn^2+^ ions (−384.76 kcal/mol for PbCl_2_ and −459.81 kcal/mol for MnCl_2_, see the Experimental Section). Also, Pb binds stronger to S (346 kJ/mol) than to Cl (301 kJ/mol), plus the formation of PbS over any Mn-based compounds might be favored by the lower lattice mismatch of PbS with CsPbCl_3._^43^ We also tested Na^+^, in the form of metal oleate (see Figures S2 and S3), since the Na–Cl bond also has strong dissociation energy (410 kJ/mol).^42^ In this case, we also observed the formation of CsPbCl_3_–PbS heterostructures, similar to the Mn^2+^ case; however, accompanied by isolated NCs of various compositions, including large CsPbCl_3_ NCs and amorphous aggregates containing mainly Na and Cl (based on EDX elemental mapping, Figure S3). Hence, Na^+^ ions are also scavenging Cl^–^ ions, but they do not seem to work as effectively as Mn^2+^ ions in terms of purity of the final product. We also need to stress that other parameters might play important roles in these reactions, such as the stabilities of the various metal–ligand complexes and the lattice energies of the various phases that are formed. All of these roles are hard to disentangle.

In this sample grown using S-ODE, a 33% Mn feed ratio, and a 220 °C reaction temperature, the CsPbCl_3_–Pb_4_S_3_Cl_2_ heterostructures were the predominant product, as evidenced by XRD, optical spectra, and TEM analysis (Figures 1e–g and S4a). The modification of this synthesis scheme, for example by increasing the Mn^2+^ feed ratio (X_Mn_ > 33.3%), did not lead to an increase in the population of PbS-based heterostructures (Figure S4) but rather to a mixture of isolated chalcohalide NCs, PbS NCs, and nonuniform heterostructures (both CsPbCl_3_–Pb_4_S_3_Cl_2_ and CsPbCl_3_–PbS), as indicated by XRD (Figure S4e). Different reaction temperatures (spanning the 135°- 250 °C window) had an impact on the size of the heterostructures, but not on their phase purity (Figures S5–S7), and indeed the XRD patterns continued to show the presence of mainly the Pb_4_S_3_Cl_2_ phase. Also, the sample showed no IR emission from PbS, most likely because the PbS domains were quite large. This unfortunately betrayed our expectations of being able to promote the almost exclusive nucleation of PbS at lower temperatures, based on our considerations on the lower formation energy of lead sulfide over that of lead chalcohalides.^44^

Switching to a more reactive sulfur source than S-ODE, namely, bis (trimethylsilyl)sulfide (TMS, see Figures 1b,c and S8–S13), led instead to phase-pure CsPbCl_3_–PbS heterostructures in the 125–200 °C temperature window. At low temperatures (125 °C), the heterostructures were small in size (∼7.2 nm) and were emitting in the NIR range (with a peak at 1250 nm, see Figures 1e and S9), indicating the formation of quantum-confined PbS. The phase purity and the dominance of PbS over Pb_4_S_3_Cl_2_ were evident from XRD (Figures 1f,g, and S9b), high-angle annular dark field (HAADF) HR STEM, and STEM-EDX (Figure S10). Bigger CsPbCl_3_–PbS heterostructures could be synthesized at higher reaction temperatures (Figures S8 and S9a). However, at reaction temperatures exceeding 140 °C, small and isolated Pb_3_S_2_Cl_2_ NCs were also formed. These could easily be removed by size-selective precipitation (Figure S11), thus recovering pure CsPbCl_3_–PbS heterostructures (Figure 1b). At higher temperatures (>160 °C), the heterostructures were more heterogeneous in size/shape (Figure S8), and their PbS-CsPbCl_3_ purity (after size-selective precipitation) remained until 180 °C, as confirmed by XRD (at 200 °C, the reflections of the Pb_4_S_3_Cl_2_ phase started to appear).

Reaction time was also an important parameter in controlling the growth of the heterostructures. Figures S12 and S13 demonstrate the heterostructure’s size tunability for two different time intervals (5 and 20 min) at three reaction temperatures (135, 140, and 150 °C). In all cases, we observed a gradual increase in heterostructure size. The aforementioned effective strategy (150 °C/TMS) to monodispersed CsPbCl_3_–PbS heterostructures may also stem from the ability of the trimethylsilyl group to act as an efficient scavenger for Cl^–^ ions. This should occur through a dehalosilylation reaction that would produce stable and volatile trimethylsilylchloride.^45^ We verified that TMS alone, in the absence of Mn^2+^, can also lead to CsPbCl_3_–PbS heterostructures, albeit contaminated with other products (Figure S14). However, the synergistic use of an exogenous cation (Mn^2+^) and TMS has proven to be the most effective approach for preparing pure PbS-based heterostructures. It is worth noting that the emission from the perovskite domain was severely quenched when it was bound to a PbS domain (Figure S15). This could be attributed to the band alignment at the heterojunction, as will be discussed later.

**Figure 2:**
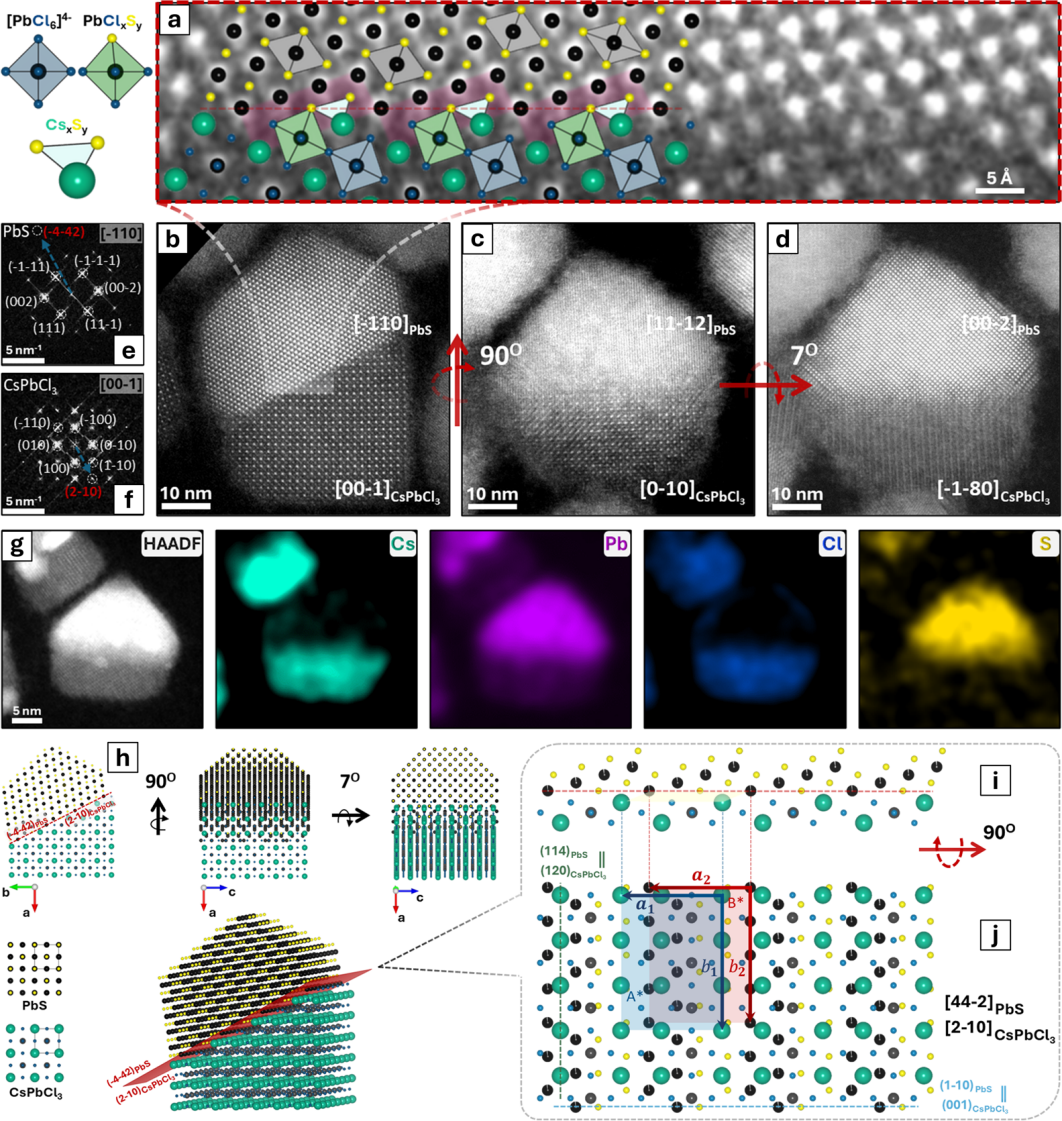
Structural and compositional analysis of CsPbCl_3_–PbS epitaxial heterostructures. (a) 3D reconstructed model superimposed on the magnified view of the stair-like epitaxial interface between CsPbCl_3_ and PbS domains (the corresponding HAADF-STEM image is reported in panel (b)). The [PbCl_6_]^4–^ octahedra in the bulk of the perovskite domain are in light blue, while the PbCl_*x*_S_*y*_ octahedra at the interface are in light green, underlining the continuity of the Pb^2+^ sublattice through coordination with S^2–^ ions. The dashed red line crossing the Pb^2+^ ions at the interface corresponds to the (−4–42) PbS and (−210) CsPbCl_3_ sets of parallel planes. (b,d) HAADF-STEM images of two heterostructures in mutually orthogonal orientations. Panel (d) is the same heterostructure as in panel (c) after a 7° tilt around an axis lying in the horizontal direction. The orthogonal projection is represented in (b). In this case, the heterostructure aligns both domains with low-index zone axes ([−110] for PbS and [00–1] for CsPbCl_3_). (e,f) FFT patterns of the corresponding PbS (e) and CsPbCl_3_ (f) domains of panel (b). Red dashed circles represent two sets of planes parallel to the interface. (g) HAADF-STEM image of a single heterostructure with the corresponding STEM-EDX elemental maps. (h) 3D model of CsPbCl_3_–PbS heterostructures shown in the same orientations as the heterostructures in panels (b–d). (i) Cross-sectional view of the interface plane. (j) Top view of the interface plane between the two domains (90° rotation around the *x*-axis from the cross-sectional view presented in (i)). Two distinct surface supercells A* and B* are identified (red and blue rectangles) for each domain, indicating minimal lattice mismatch (<0.4% for a and b).

The epitaxial relationships between the two domains of the CsPbCl_3_–PbS heterostructure were investigated by HAADF-STEM. Figure 2 reports atomic resolution images of single heterostructures in which the CsPbCl_3_ and PbS domains share a sharp interface. Several projected views of the heterostructure are accessible: for example, two different CsPbCl_3_–PbS NCs are shown here (Figure 2b,c) in mutually orthogonal projections. In one of them (Figure 2b), both domains present low-index zone axes ([00–1] for CsPbCl_3_ and [−110] for PbS), and the atomic columns are visible, enabling atomic resolution imaging of the interface. In the other projection (Figure 2c), only the perovskite domain is on a low-index zone axis [0–10], and the atomic columns in the PbS domain are not clearly resolved, as it is viewed from a high-index direction. However, by tilting the sample by 7° around an axis lying in the horizontal direction, the [00–2] zone axis could be accessed (Figure 2d), allowing us to identify the periodicity and rock-salt cubic crystal structure of the PbS domain. The mutual orthogonality of the projections displayed in Figure 2b,c was further confirmed by FFT analysis of the corresponding STEM images (Figures 2e,f and S16). Using the extracted crystallographic information, we constructed a 3D model of the heterostructure. This model, reported in Figure 2h, closely matches the atomic columns of each heterostructure projection, as represented in the magnified images (Figures 2a and S16). A closer look at the interface revealed a stair-like repetitive motif between the two domains, as depicted in Figure 2a,i. Such interface exposes multiple sets of parallel planes at the junction. The interface plane is depicted by a dashed red line in Figure 2a,h,i. It consists of the (−4–42) PbS and (2−10) CsPbCl_3_ sets of parallel planes of the respective domains. The directions of these crystallographic planes can be inferred from the corresponding FFT patterns (Figures 2e,f) illustrating their parallel alignment along the interface. Figures 2i,j are magnified and rotated views of the interface, so that it is either seen in side-view (Figure 2i) or top-view (Figure 2j, that is, along the vector perpendicular to the interfacial plane).

We marked two distinct surface supercells A* and B* (red and blue rectangles) and their corresponding lattice vectors  and , and  and , where *g* is the unit vector of the corresponding crystallographic direction, for each crystal. These are repetitive two-dimensional unit cells of each phase that can reproduce the crystal lattice at the interface by translation. The slight difference in dimensions of the two cells suggests a minimal lattice mismatch (less than 0.4% for *a* and *b*), which is a crucial factor for isostructural phases to establish a favorable epitaxial relationship. Elemental analysis by STEM-EDX confirmed a homogeneous distribution of the constituent elements within the two distinct domains of a single heterostructure (Figure 2g) as well as its stoichiometry.

**Figure 3:**
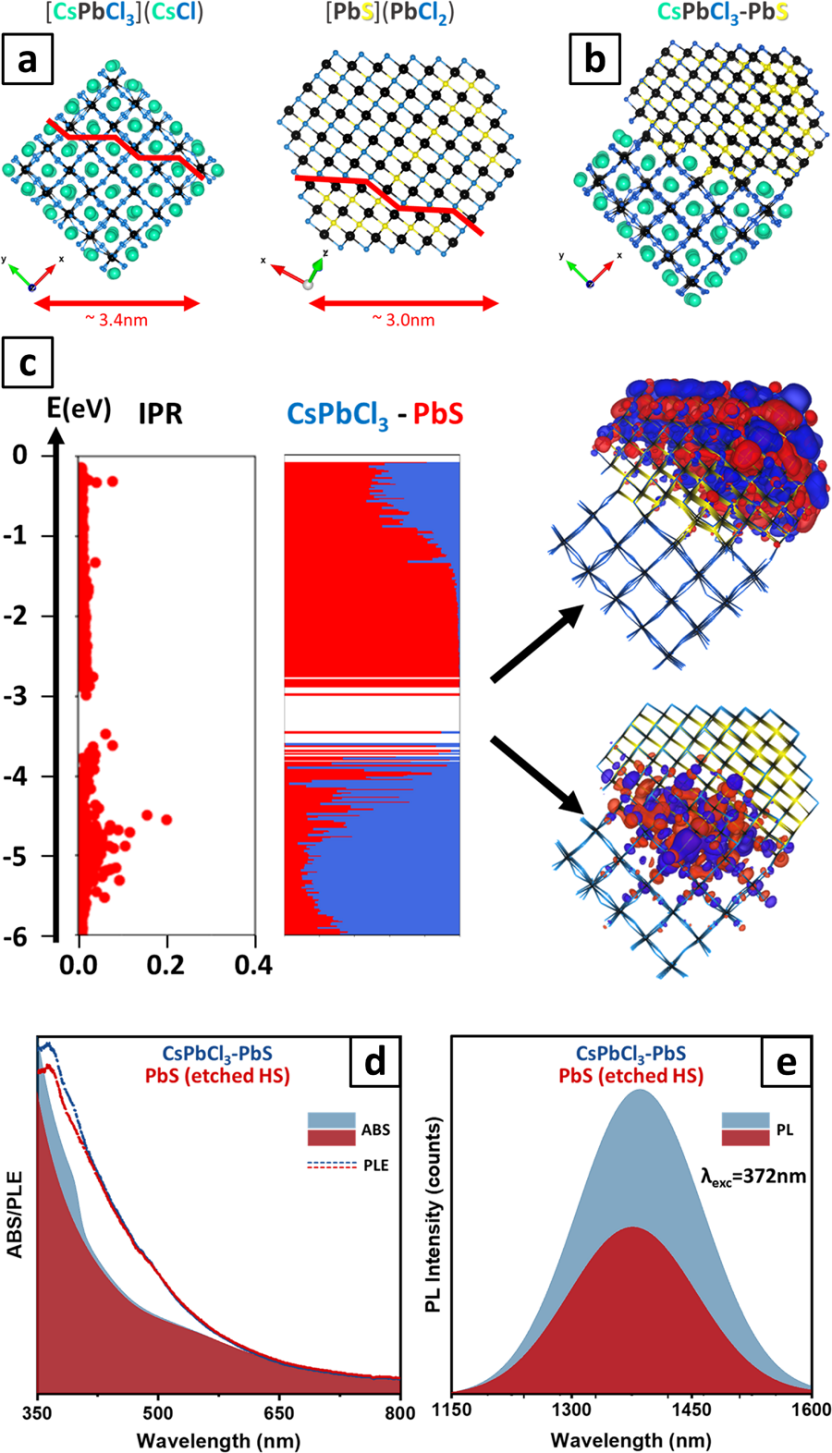
Computational and optical properties. (a) Ball and stick representation of (left) a cubic ∼3.4 nm-sided CsPbCl_3_ NC model and (right) a cuboctahedral ∼3.0 nm-sided PbS NC model optimized at the DFT/PBE level of theory. (b) Preparation of the heterostructure NC model by cutting the PbS and CsPbCl_3_ models along the planes evidenced as red lines in (a) and stacking along the [110] axis to fit the experimental STEM images. (c) IPR plot and electronic structure of the CsPbCl_3_/PbS NC heterostructure model computed at the DFT/PBE level of theory. The color code indicates the contribution of each domain to each molecular orbital. On the right are plotted the frontier molecular orbitals. (d) Optical absorption (colored curves) and PL excitation (dashed lines) spectra for both CsPbCl_3_/PbS heterostructures and PbS NCs recovered after etching away the CsPbCl_3_ domain. (e) PL spectra of the same samples, using an excitation wavelength of 370 nm.

Aided by the microscopy analysis discussed above, we performed atomistic calculations at the DFT level to better understand the structural features of the CsPbCl_3_/PbS heterostructures, particularly focusing on the interface between the two domains. We started by preparing two separate models of CsPbCl_3_ and PbS NCs in shapes that would match the two separate domains if allowed to grow individually as single NCs, i.e., respectively as a cube for CsPbCl_3_ and a cuboctahedron for PbS. These are illustrated in Figure 3a. The cubic CsPbCl_3_ NC model of about 3.4 nm on each side was essentially the same as the CsPbBr_3_ NC models widely reported in the literature, exhibiting six (001) facets.^46,47^ The structure of this model was relaxed at the DFT level. Concomitantly, a PbS NC model of about 3.0 nm was prepared by carving a bulk rock-salt structure to prepare a NC featuring six stoichiometric (100) facets and eight anion-rich (111) facets. In this case, the PbS structure was relaxed at the DFT level, as well. In both models, Cl^–^ anions were employed as a convenient way to simulate surface ligands while containing the computational effort and preventing complications in the analysis. As illustrated in Figure 3a, to prepare the heterostructure, we first oriented the CsPbCl_3_ model along the [001] axis and cut the (110) planes in a stair-like fashion to reproduce the experimental pattern of Figure 2b–d. We then aligned the PbS NC model along the [110] axis and cut the (111) planes in a stair-like fashion (with a repetition unit of four Pb cations) to match the experimental STEM image of Figure 2b. Finally, we made the two fragments fit together to obtain a CsPbCl_3_/PbS NC model featuring a charge-balanced Cs_126_Pb_337_S_221_Cl_358_ stoichiometry, as depicted in Figure 3b. Importantly, the stair-like epitaxial alignment enables complete coordination of the Pb^2+^ cations at the interface of the PbS and CsPbCl_3_ domains, where the anions (either Cl^–^ or S^2–^) fill the halide vacancies of the perovskite fragment. This compensation results in a heterostructure without overall halide vacancies, leading to a smooth relaxation of the final model at the DFT level.

Based on this model, we computed the electronic structure and performed an inverse participation ratio (IPR) analysis to evidence the presence of localized states in the electronic structure (IPR values deviating significantly from zero), as presented in Figure 3c.^48^ The computed heterostructure features a clean band gap, free of midgap states: here, the conduction band edge states are mainly delocalized over the PbS domain, whereas the valence-band edge states, also dominated by the contribution of the PbS domain, present intermixed contributions of the two domains and are more localized at the interface.

We also directly compared the optical properties of CsPbCl_3_/PbS heterostructures with those of the PbS NCs recovered from them after etching away the CsPbCl_3_ domain (see the Experimental Section for details). XRD and TEM analyses of this sample indicated pure PbS (Figures S17 and S18). The absorption spectra of the two samples are almost superimposable, except for the feature in the UV region for the heterostructure sample ascribed to the perovskite domain (Figure 3d). The PL in the NIR region from the PbS sample was less intense and slightly blue-shifted compared to that of the heterostructure sample (Figure 3e; both samples had the same optical density at 500 nm). We may attribute the blue shift either to a slightly smaller PbS size resulting from the removal of surface layers during etching (Figure S18) or to a slightly stronger confinement of the carriers in freestanding PbS compared with PbS interfaced with CsPbCl_3_. Photoluminescence excitation (PLE) spectra for both samples (recorded at 1380 nm; Figure 3d) closely resembled their respective absorption curves. This, for the heterostructure, indicates that the NIR emission may also originate from the carriers photogenerated in the CsPbCl_3_ domain and transferred from there to the PbS domain.

**Figure 4:**
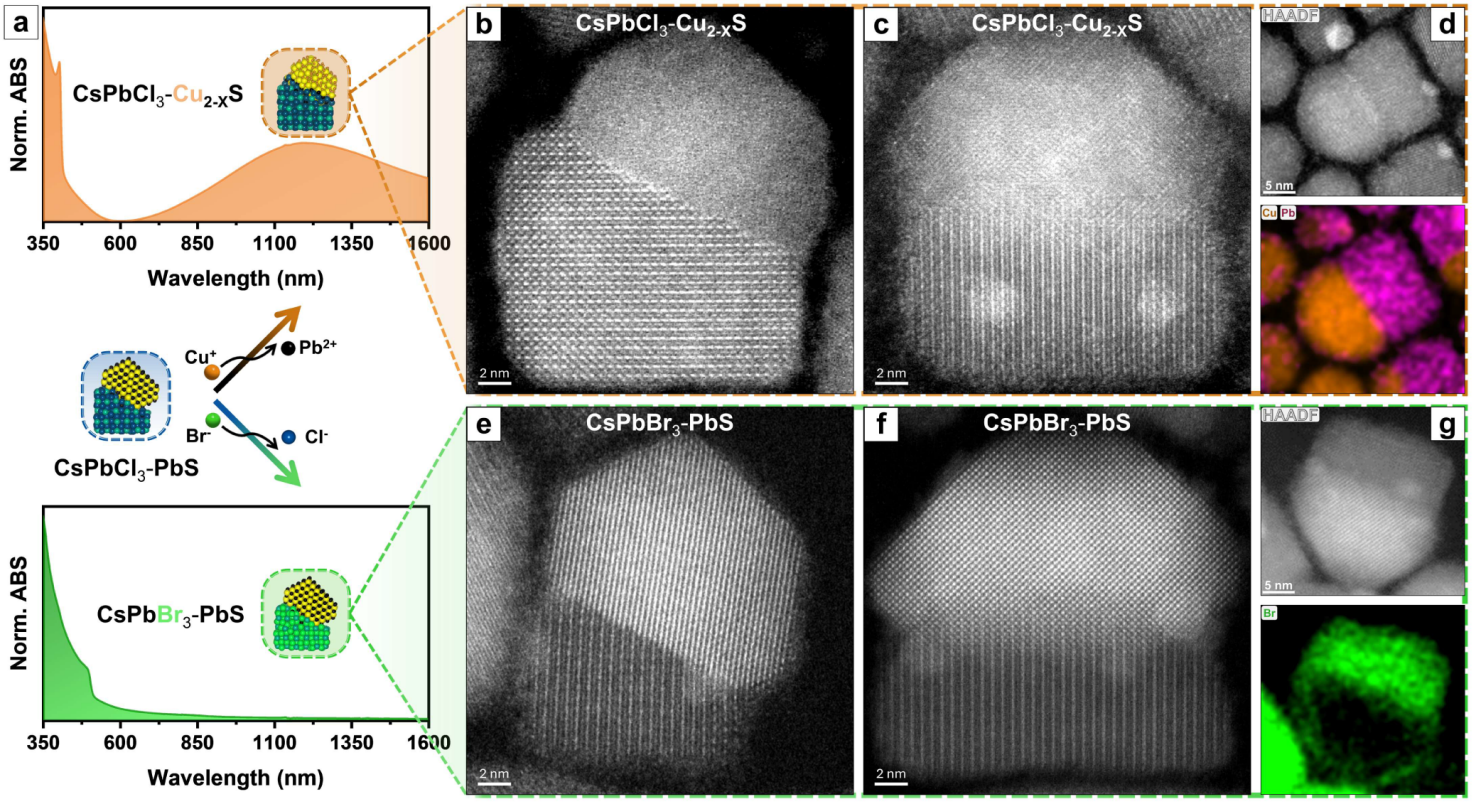
Selective cation (Pb^2+^→ Cu^+^) and anion (Cl^–^→ Br^–^) exchange on the CsPbCl_3_/PbS heterostructure. (a) Optical absorption spectra of cation (Pb^2+^ → Cu^+^)-exchanged CsPbCl_3_/Cu_2-*x*_S (upper) and anion (Cl^–^ → Br^–^)-exchanged CsPbBr_3_/PbS heterostructures (down). (b,c) High-resolution HAADF-STEM images of two different cation-exchanged CsPbCl_3_/Cu_2-*x*_S heterostructures in common orthogonal projections. (d) HAADF-STEM image of a single CsPbCl_3_/Cu_2-*x*_S heterostructure with the corresponding STEM-EDX elemental map for Pb^2+^ (purple) and Cu^+^ (orange). (e,f) HAADF HR-STEM images of two different anion-exchanged CsPbBr_3_/PbS heterostructures in orthogonal projections. (g) HAADF-STEM image of a single CsPbBr_3_/PbS heterostructure with the corresponding STEM-EDX elemental map for Br^–^ (green).

**Figure 5:**
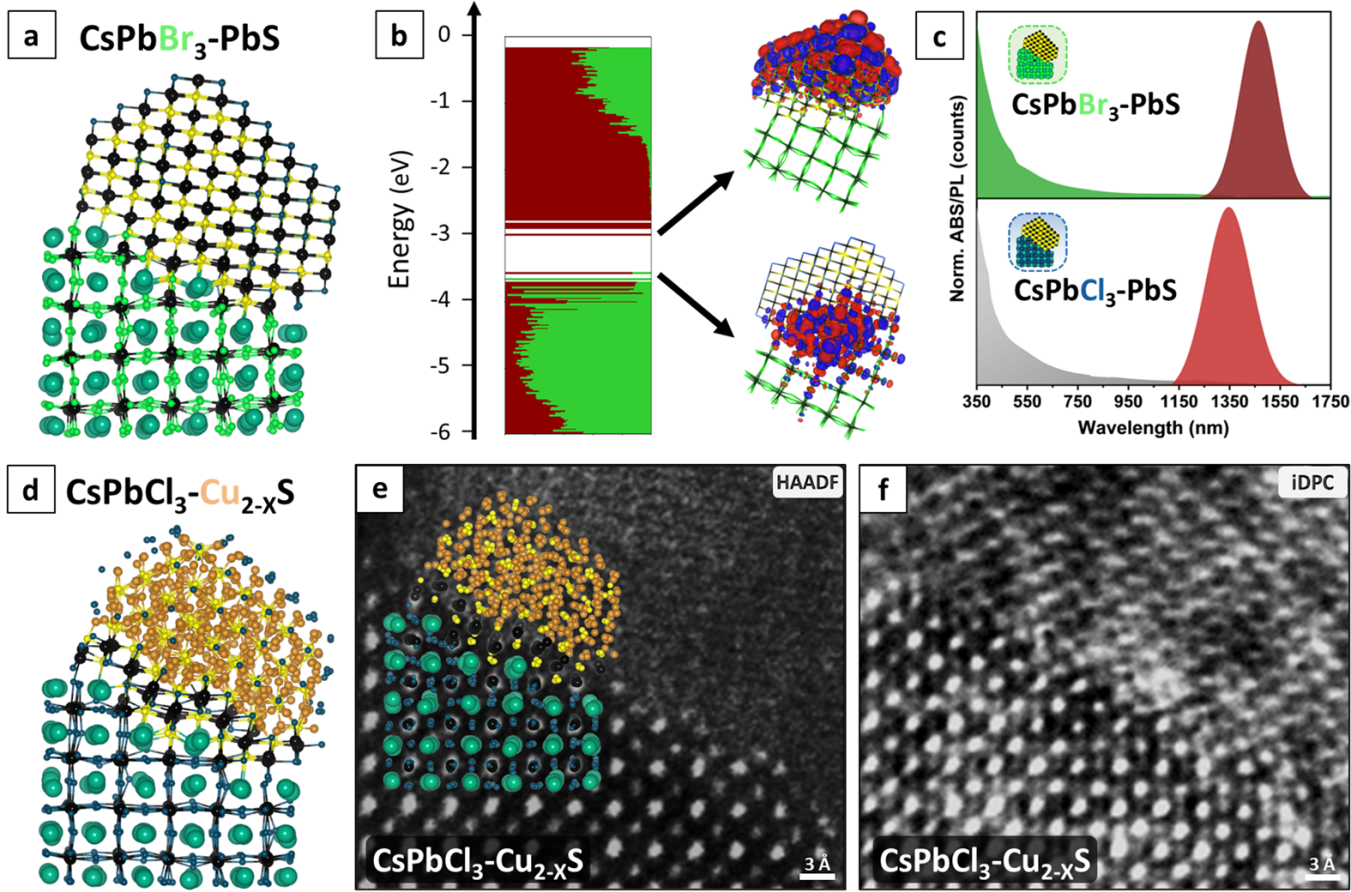
(a) Ball and stick representation of the CsPbBr_3_/PbS NC heterostructure model optimized at the DFT/PBE level of theory. (b) The electronic structure of the model represented in (a) is computed at the DFT/PBE level of theory. The color code indicates the contribution of each domain to each molecular orbital. On the right, we plotted the frontier molecular orbitals to further highlight the resemblance with the parent CsPbCl_3_/PbS NC. (c) Optical absorption and PL spectra of pure CsPbCl_3_/PbS (down panel, sample “150 °C/TMS”), and the corresponding anion (Cl^–^ → Br^–^) exchanged CsPbBr_3_/PbS heterostructures (upper panel). (d) Ball and stick representation of the CsPbCl_3_/Cu_2_S NC heterostructure model optimized at the DFT/PBE level of theory. (e) High-resolution HAADF-STEM image of cation-exchanged CsPbCl_3_/Cu_2-*x*_S highlighting the stair-like epitaxial interface. The superimposed DFT model confirms the presence of a PbS monolayer at the interface. (f) iDPC STEM image of the same area highlighting atomic columns of light elements (Cl, Cu, and S).

Postsynthesis ion exchange reactions in colloidal NCs have been established as an effective strategy to access a wide range of nanomaterials, including alloys or heterostructures, which are challenging to prepare with direct synthesis methods.^49−52^ In a similar fashion, the CsPbCl_3_/PbS heterostructures may be used as templates for postsynthesis ion exchange reactions in both domains of the dimer. This would enable the selective modification of the composition of each domain. As a demonstration of this versatile process, we exposed the CsPbCl_3_/PbS heterostructures to either Br^–^ or Cu^+^ ions. This allowed us to induce either full anion exchange in the perovskite domain or full cation exchange in the metal sulfide domain, resulting in the formation of two possible daughter heterostructures, CsPbBr_3_/PbS and CsPbCl_3_/Cu_2-*x*_S. Such exchanges were carried out using two different samples of the CsPbCl_3_/PbS heterostructure, i.e., those previously named “150 °C/TMS” and “220 °C/S-ODE” (see Figure 1). The former sample, as previously discussed, consisted of small, pure CsPbCl_3_/PbS heterostructures, and allowed an easier investigation of the crystal phase (Figure S19) and optical properties (Figures 5c and S20) after the exchange. The latter sample consisted instead of bigger (albeit less pure) CsPbCl_3_/PbS heterostructures, to facilitate their microscopy study (Figures 4, S21, and S23). Starting from the “150 °C/TMS” sample, both exchanges were confirmed by modifications in the optical spectra and XRD patterns (Figures S19, 5c, and S20). The Br-exchanged sample presented the characteristic excitonic absorption of the CsPbBr_3_ perovskite phase while maintaining the IR absorption of the PbS domain (Figure 5c).

The PLE spectrum (Figure S22) of the CsPbBr_3_–PbS heterostructures closely resembles their absorption spectrum (Figure 5c). This similarity, observed in the parent heterostructures as well, suggests that photoexcited carriers in the CsPbBr_3_ domain get transferred to the PbS domain and contribute to the NIR emission from PbS. Cation exchange of the same samples with Cu^+^ revealed instead a broad absorption feature in the NIR spectral range, previously assigned to a localized surface plasmon resonance (Figure S20a)^51,53^ and attributed to the substoichiometric composition of the digenite (*Fm*3̅*m*) Cu_2-*x*_S phase (Figure S19). Also, the Cu^+^-treated heterostructure lost the IR emission originally stemming from the PbS domain. Such exchanges on the “220 °C/S-ODE” sample resulted in similar observations. For instance, the Br^–^ exchange led to a red-shifted perovskite excitonic absorption (Figure 4a, lower panel), whereas the Cu^+^ treatment yielded a broad absorption in the NIR range (Figure 4a, upper panel).

The distribution of the ions in the exchanged heterostructure samples was investigated by STEM-EDX. Compositional mapping of a single heterostructure treated by Cu^+^ indicates pronounced segregation of Pb^2+^ and Cu^+^ ions within the two distinct domains of the heterostructure, CsPbCl_3_ and Cu_2-*x*_S, respectively (Figure 4d). This observation implies complete conversion from PbS to Cu_2-*x*_S while retaining the integrity of the anionic framework, suggesting a heterostructure with CsPbCl_3_/Cu_2–*x*_S composition. Additionally, elemental maps of a single CsPbBr_3_/PbS heterostructure evidenced a homogeneous distribution of Br^–^ ions throughout the perovskite domain (Figure 4g). EDX agrees with the XRD patterns and the optical measurements, confirming full preservation of the PbS domain after anion exchange. Even though neither Cs^+^ nor Br^–^ were present inside the PbS domain, both ions could be detected on its surface (Figure S23).

HAADF-STEM analyses of the exchanged heterostructures also revealed similar epitaxial relationships with the parent CsPbCl_3_–PbS heterostructure, since the FFT patterns demonstrate identical orientations with the constructed 3D model (Figure S23). This evidence highlights that the exchange did not disrupt the overall structure, proving that the anionic and cationic frameworks remained intact during the reactions. We identified the orientations and parallel planes for CsPbCl_3_/Cu_2–*x*_S through FFT analysis using two orthogonal projections of the heterostructure (Figure 4b,c). Figure 4b illustrates an atomic resolution image of the Cu_2-*x*_S–CsPbCl_3_ heterostructure in low-index zone axes ([001] CsPbCl_3_ and [1–10] Cu_2-*x*_S). The corresponding FFTs show that the (−4–42) Cu_2-*x*_S and (2–10) CsPbCl_3_ planes are parallel and along the interface. These two sets of planes were also found to constitute the interface in the case of the parent CsPbCl_3_-PbS heterostructure. The FFT patterns (Figure S23f,g) indicate that the zone axes of the first projection (Figure 4b) are parallel planes in the second projection (Figure 4c), confirming the orthogonality of the two projections. A more careful analysis through STEM-EDX, HAADF-STEM, and integrated differential phase contrast (iDPC) STEM, as presented in Figures 5e,f, and S24, revealed the presence of a PbS monolayer between the CsPbCl_3_ and Cu_2-*x*_S domains. The resistance of this layer to the cation exchange reaction hints at comparatively higher stability, most likely because it is located at the interface (an aspect that will be discussed in more detail later). Data related to the CsPbBr_3_/PbS heterostructure obtained by Cl^–^ → Br^–^ exchange are presented in Figure 4e–g. HAADF-STEM analyses of such cases unveiled a distinct interface between the two domains (Figure 4e,f), with the same epitaxial relationship observed in the CsPbCl_3_/PbS case. This was further confirmed by the corresponding FFT patterns, evidencing unexchanged orientations and planes of the interface (Figure S23).

The NIR emission of the exchanged CsPbBr_3_/PbS heterostructure was red-shifted with respect to the parent CsPbCl_3_/PbS samples (Figure 5c). This red shift might point to a lower degree of confinement of the carriers in PbS for the CsPbBr_3_/PbS heterostructures compared to the CsPbCl_3_/PbS case. To further shed light on the heterostructures obtained by ion exchange reactions, we performed DFT calculations on the CsPbBr_3_/PbS and CsPbCl_3_/Cu_2_S NC models. For the CsPbBr_3_/PbS heterostructure, we simply took the previously built CsPbCl_3_/PbS model and systematically replaced the Cl^–^ ions of the perovskite domain with Br^–^ ions while preserving the cation sublattice. The relaxed structure of this model is depicted in Figure 5a. As in the chloride-based heterostructure, the staircase-like epitaxial alignment of the CsPbBr_3_/PbS NC ensures a six-fold coordination of the Pb^2+^ cations at the interface between the perovskite and sulfide moieties. The electronic structure of this CsPbBr_3_/PbS model, reported in Figure 5b, is closely aligned with that of the parent CsPbCl_3_/PbS, with the main difference being a higher contribution of the Br-based perovskite to the valence band edge states, in line with a smaller band gap of CsPbBr_3_ compared to that of CsPbCl_3_. Overall, we see a slight blue shift of the bandgap of about 0.1 eV compared to CsPbCl_3_/PbS, contrary to the experimentally observed red shift. However, the exact emission energy of the PbS moiety is difficult to discern due to the presence of interfacial states that introduce additional electronic levels within the bandgap, complicating the electronic structure landscape and hindering a more precise assignment of the emission characteristics of the PbS component.

To build the CsPbCl_3_/Cu_2_S heterostructure, we also started from the parent CsPbCl_3_/PbS model and preserved the perovskite domain as well as one PbS layer at the interface (identified via HAADF-STEM and iDPC-STEM analyses), while replacing each Pb^2+^ ion with two Cu^+^ ions in the remaining PbS domain. The latter operation was facilitated by the resemblance of the cubic bulk PbS and Cu_2_S crystallographic structures, which belong to the same *Fm*3̅*m* space group and share a common S^2–^ anion sublattice. As anticipated and further evidenced in the relaxed structure of Figure 5d, the presence of an intermediate PbS layer results in a smooth transition between the two domains as it allows the completion of the coordination of the ions of both CsPbCl_3_ and Cu_2_S domains at the interface. The electronic structure of the CsPbCl_3_-Cu_2_S heterostructure, reported in Figure S25, exhibits no bandgap, mostly due to the presence of surface defects and an underestimation of the band gap typical of some of the DFT exchange-correlation functionals, like the one employed here.^54^ We also point out that the model of CsPbCl_3_-Cu_2_S heterostructures was prepared with a stoichiometric Cu_2_S composition, while the experiments revealed Cu_2-*x*_S-based heterostructures, with the presence of Cu^+^ vacancies, as seen from the absorption band in the NIR from that sample.

## Conclusions

We have reported the synthesis of epitaxial CsPbCl_3_-PbS heterostructures and analyzed their structural and optical features. The synthesis was made possible by introducing conditions that favor the nucleation and growth of PbS on top of the perovskite domain compared to the competing process of Pb_4_S_3_Cl_2_ growth. This heterostructure was then used to generate additional heterostructures by selective anion–cation exchange on either the perovskite or the lead sulfide domain. While this is proven here just for Cl^–^ → Br^–^ and Pb^2+^ → Cu^+^ exchanges, the method should be extendable to encompass other reactions. This proof-of-concept study paves the way to a vast playground for potential new structures: these will likely include the replacement of the Cs^+^ cation in the perovskite domain with other large monovalent cations such as methylammonium and formamidinium, and the Pb^2+^ cation in the PbS domain with cations such as Ag^+^, Zn^2+^, Cd^2+^, and many others, leading to heterostructures that might find applications in fields such as photocatalysis and photodetection.

## Experimental Section

### Materials

Cesium carbonate (Cs_2_CO_3_, 99.9%), lead chloride (PbCl_2_, >98%), lead bromide (PbBr_2_, >98%), lead acetate trihydrate (Pb(CH_3_COO)_2_·3H_2_O, 99.99%), manganese(II) acetate (Mn(CH_3_COO)_2_, 98%), sodium acetate (C_2_H_3_NaO_2_, 99%), copper(I) oxide (Cu_2_O, >99.9%), dodecanethiol (DDT, 99.9%), bis(trimethylsilyl)sulfide ((TMS)_2_S, C_6_H_18_SSi_2_), elemental sulfur (S, >99%), 1-octadecene (ODE, C_18_H_36_, 90%), oleic acid (OA, C_18_H_34_O_2_, 90%), oleylamine (OLAm, C_18_H_37_N, 70%), toluene (C_7_H_8_, >99.8%), ethyl acetate (C_4_H_8_O_2_), acetone (CH_3_COCH_3_, >99.5%), dimethyl sulfoxide ((CH_3_)_2_SO, >99.7%), tetrachloroethane (TCE, CHCl_2_CHCl_2_, >98%), fumed silica powder (SiO_2_), and hexane (99.8%) were purchased from Merck. All chemicals were used without further purification.

### Precursor Solution Preparation

#### PbCl_2_ Stock Solution

PbCl_2_ (2 mmol), 30 mL of octadecene, 5 mL of oleylamine, and 5 mL of oleic acid were loaded into a 100 mL three-neck flask. The mixture was first degassed for 30 min at room temperature and then for 30 min at 110 °C under stirring. Finally, the solution was heated to 150 °C under N_2_ until the salt was completely dissolved. The resulting solution was transferred into an N_2_-filled glass vial and stored inside a glovebox for further use.

#### Cs-OL Stock Solution

Cs_2_CO_3_ (1 mmol), 2.5 mL of oleic acid, and 8.75 mL of octadecene were loaded into a 25 mL three-neck flask. The mixture was first degassed for 30 min at room temperature and then for 1 h at 110 °C under stirring. The resulting solution was transferred into an N_2_-filled glass vial and stored inside a glovebox for further use.

#### Pb-OL Stock Solution

1 mmol of lead acetate trihydrate, 650 μL of oleic acid, and 9.35 mL of octadecene were loaded into a 50 mL three-neck flask. The mixture was first degassed for 30 min at room temperature and then for 1 h at 110 °C under stirring. The resulting solution was transferred into an N_2_-filled glass vial and stored inside a glovebox for further use.

#### Mn-OL Precursor Solution

0.2 mmol of manganese acetate, 650 μL of oleic acid, and 9.35 mL of octadecene were loaded into a 50 mL three-neck flask. The mixture was first degassed for 30 min at room temperature and then for 1 h at 110 °C under stirring. The resulting solution was transferred into an N_2_-filled glass vial and stored inside a glovebox for further use.

#### Na-OL Precursor Solution

0.1 mmol of sodium acetate, 650 μL of oleic acid, and 9.35 mL of octadecene were loaded into a 50 mL three-neck flask. The mixture was first degassed for 30 min at room temperature and then for 1 h at 110 °C under stirring. The resulting solution was transferred into an N_2_-filled glass vial and stored inside a glovebox for further use.

#### S-ODE Precursor Solution

0.5 mmol of sulfur powder and 5 mL of ODE (previously degassed for 1 h at 110 °C) were loaded into a 7 mL vial inside a glovebox. The resulting mixture was sonicated until the sulfur powder was completely dissolved.

#### TMS-ODE Precursor Solution

0.5 mmol of TMS and 5 mL of ODE (previously degassed for 1 h at 110 °C) were loaded and mixed in a 7 mL vial inside a glovebox.

#### Cu-OL Stock Solution (Cation Exchange Reactions)

0.1 mmol of Cu_2_O, 650 μL of oleic acid, and 9.35 mL of octadecene were loaded into a 40 mL vial. The mixture was degassed first for 30 min at room temperature and then for 1 h at 110 °C under stirring. The resulting solution was transferred into an N_2_-filled glass vial and was stored inside a glovebox for further use.

#### PbBr_2_ Stock Solution (Halide Exchange Reactions)

1 mmol of PbBr_2_, 2.5 mL of oleic acid, 2.5 mL of oleylamine, and 15 mL of octadecene were loaded into a 40 mL vial. The mixture was degassed for 30 min at room temperature and then for 30 min at 110 °C. Then, the solution was heated up to 150 °C under N_2_ until the salt was completely dissolved. The resulting solution was transferred into an N_2_-filled glass vial and stored inside a glovebox for further use.

### Nanocrystal Synthesis

#### CsPbCl_3_ Cluster Synthesis

CsPbCl_3_ nanoclusters were synthesized following a previously reported method with slight modifications.^41^ Briefly, 4 mL of the PbCl_2_ stock solution was transferred to a 20 mL vial inside a glovebox. The solution was heated up to 50 °C and then 0.25 mL of Cs-OL stock solution was injected into the PbCl_2_ stock solution. The resulting mixture was stirred at 50 °C for approximately 30 min. After this time, the appearance of a white-turbid color indicated the assembly of CsPbCl_3_ cluster formation. The resulting solution was purified by centrifugation (8000 rpm, 5 min), and the supernatant was discarded. Finally, the precipitate was redispersed in a solution containing 0.9 mL of previously degassed octadecene and 0.3 mL of Mn-OL (or Na-OL) solution.

#### CsPbCl_3_–PbS Heterostructure Synthesis

In a typical synthesis, 4.0 mL of previously degassed octadecene was loaded into a 20 mL N_2_-filled vial. Then, the vial was heated to the corresponding reaction temperature (125–220 °C) for 5 min. After this time, the following precursor solutions were swiftly injected into octadecene: 0.1 mL of Pb-OL solution, 0.2 mL of DDT-ODE solution, cluster solution (mentioned above), and 0.1 mL of the sulfur source (S-ODE for large CsPbCl_3_–PbS heterostructures (“220 °C/S-ODE” synthesis) or TMS-ODE for small and pure CsPbCl_3_–PbS heterostructures (“150 °C/TMS” synthesis)). The reaction mixture was kept under stirring for the corresponding reaction time (3–20 min) and finally quenched in an ice–water bath. The heterostructures were purified by centrifugation (6000 rpm, 5 min); the supernatant was discarded, and the precipitate was redispersed in toluene. For concentration reference, the absorption spectrum of the NC solution was measured (after a dilution of 50 μL of NCs to 2.5 mL of toluene), presenting an optical density of 0.26 at 370 nm.

### Exchange Reactions

#### Cu-Exchange Reactions

Cation exchange reactions were performed in a glovebox. In a typical reaction, 750 μL of the CsPbCl_3_–PbS solution and 500 μL of Cu-OL stock solution were loaded into a 7 mL vial. The reaction was kept overnight under stirring at room temperature. Afterward, the heterostructures were purified with EtOAc (600 μL) and collected by centrifugation (6000 rpm, 5 min). The supernatant was discarded, and the precipitate was redispersed in toluene for further characterization.

#### Br-Exchange Reactions

Halide exchange reactions were performed in a glovebox. In a typical reaction, 750 μL of the CsPbCl_3_–PbS solution and 500 μL of the PbBr_2_ stock solution were loaded into a 7 mL vial. The reaction was kept overnight under stirring at room temperature. Then, the heterostructures were purified with EtOAc (600 μL) and collected by centrifugation (6000 rpm, 5 min). The supernatant was discarded, and the precipitate was redispersed in toluene for further characterization.

#### CsPbCl_3_–PbS Heterostructure Etching

PbS NCs were obtained from the etching of the CsPbCl_3_–PbS heterostructures. The CsPbCl_3_ domain was etched from the heterostructures by dissolving it in a mixture of CsPbCl_3_–PbS (1 mL), DMSO (0.25 mL), and acetone (0.25 mL). The resulting PbS NCs were collected by centrifugation (6000 rpm for 5 min) and redispersed in toluene.

#### Optical Characterization

UV–vis absorption spectra were obtained using a Varian Cary 5000 UV–vis-NIR absorption spectrophotometer (Agilent). The spectra were collected by diluting 50 μL of the sample in toluene in 2.5 mL of TCE. UV–vis photoluminescence spectra were obtained on a Varian Cary Eclipse spectrophotometer (Agilent) using λ_ex_ = 370 nm. IR photoluminescence spectra were obtained on an Edinburgh FS5 spectrofluorometer using λ_ex_ = 370 nm. UV–vis-NIR excitation spectra were obtained on an Edinburgh FS5 spectrofluorometer. The experimental emission spectra were fitted (Gauss fit) to remove noise in the data in the NIR spectral region.

#### Transmission Electron Microscopy

Bright-field TEM images of the samples were acquired with a JEOL-1400Plus transmission electron microscope operating at an acceleration voltage of 100 kV and on a JEOL JEM 1011 transmission electron microscope operating at an acceleration voltage of 120 kV. Samples were prepared by drop casting from diluted NC solutions onto carbon-film-coated 200 mesh copper grids.

#### X-Ray Powder Diffraction

X-ray powder diffraction measurements were performed on a PANalytical Empyrean X-ray diffractometer equipped with a 1.8 kW Cu Kα ceramic anode and a PIXcel3D 2 × 2 area detector, operating at 45 kV and 40 mA. NCs’ dispersions were mixed with fumed silica and dried to minimize the preferential orientation. Finally, the powder was placed on a zero-diffraction silicon substrate to perform the measurements.

#### Scanning Transmission Electron Microscopy (STEM) Characterization

High-resolution scanning TEM (HRSTEM) images were acquired on a probe-corrected ThermoFisher Spectra 30–300 S/TEM operated at 300 kV, using a HAADF detector with a beam current of a few tens of picoamperes to limit beam damage to the sample. The iDPC images were collected on a segmented Panther detector; iDPC, compared with HAADF, provides more contrast for light elements. The convergence angle was set to 25 mrad, corresponding to a subangstrom electron beam. Compositional maps were acquired using Velox, with a probe current of ∼150 pA and rapid rastered scanning. The energy-dispersive X-ray (EDX) spectroscopy signal was acquired on a Dual-X setup comprising two detectors on either side of the sample, for a total acquisition solid angle of 1.76 Sr.

#### Computational Methodology

To shed light on the structural and electronic properties of the CsPbCl_3_–PbS, CsPbBr_3_–PbS, and CsPbCl_3_–Cu_2_S heterostructured NCs, we carried out atomistic simulations at the density functional theory (DFT) level using the PBE exchange–correlation functional^55^ and a double-ζ basis set plus polarization functions on all atoms^56^ as implemented in the CP2K 6.1 package.^57^ Scalar relativistic effects were incorporated as effective core potential functions in the basis set.^56^ All structures were optimized in a vacuum. Details on how the models were prepared are reported in the main text. For the parent CsPbCl_3_–PbS, we have additionally computed the inverse participation ratio (IPR)^58^ in order to identify surface-localized states. As demonstrated for other NCs,^48^ the IPR quantifies the orbital localization of a given molecular orbital and is defined as
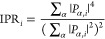


Here, *P*_α,*i*_ represents the weight of the molecular orbital *i* on a given atom α expanded on an atomic orbital basis. For finite systems, the IPR provides an estimate of the number of atoms that contribute to a given electronic state *i*. It can range from the inverse of the number of atoms in the system when the wave function is distributed equally over all atoms in the system to 1 in the case of a localized state to a single atom. When values are near zero, the IPR identifies delocalized states.

To verify that the presence Mn^2+^ ions could modify the fraction of the available Cl^–^ ions in solution, we also computed the binding affinity of MnCl_2_ and PbCl_2_, defined as
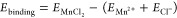
where the MnCl_2_ molecular complex structures have been relaxed in a vacuum at the DFT/PBE/DZVP level of theory. The Mn^2+^ ions were considered in an open-shell sextuplet electron configuration.

## Supporting Information Available

Control syntheses; stability tests; additional data on optical, structural, morphological, and compositional characterization (PDF)
