# Supplementary material for: Nanocrystal Heterostructures Based on Halide Perovskites and Metal Sulfides
Source: J Am Chem Soc. 2024 Sep 30;146(40):27571–82. doi: 10.1021/jacs.4c08565 (PMC11467908; doi:10.1021/jacs.4c08565)
Supplement: Supplementary file 1 — ja4c08565_si_001.pdf [file ja4c08565_si_001.pdf]

Supporting Information for

Nanocrystals Heterostructures based on Halide Perovskites and Metal Sulfides

Nikolaos Livakas[1,2], Juliette Zito[1], Yurii P. Ivanov[3], Clara Otero-Martínez[4], Giorgio Divitini[3*], Ivan Infante[5,6*], Liberato Manna[1*]

[1] Nanochemistry, Istituto Italiano di Tecnologia, Via Morego 30, Genova, Italy

[2] Dipartimento di Chimica e Chimica Industriale, Università di Genova, 16146 Genova, Italy

[3] Electron Spectroscopy and Nanoscopy, Istituto Italiano di Tecnologia, Via Morego 30, Genova, Italy

[4] CINBIO, Department of Physical Chemistry, Materials Chemistry and Physics Group, Universidade de Vigo, Campus Universitario As Lagoas-Marcosende, 36310 Vigo, Spain

[5] BCMaterials, Basque Center for Materials, Applications, and Nanostructures, UPV/EHU Science Park, Leioa 48940, Spain

[6] Ikerbasque Basque Foundation for Science Bilbao 48009, Spain

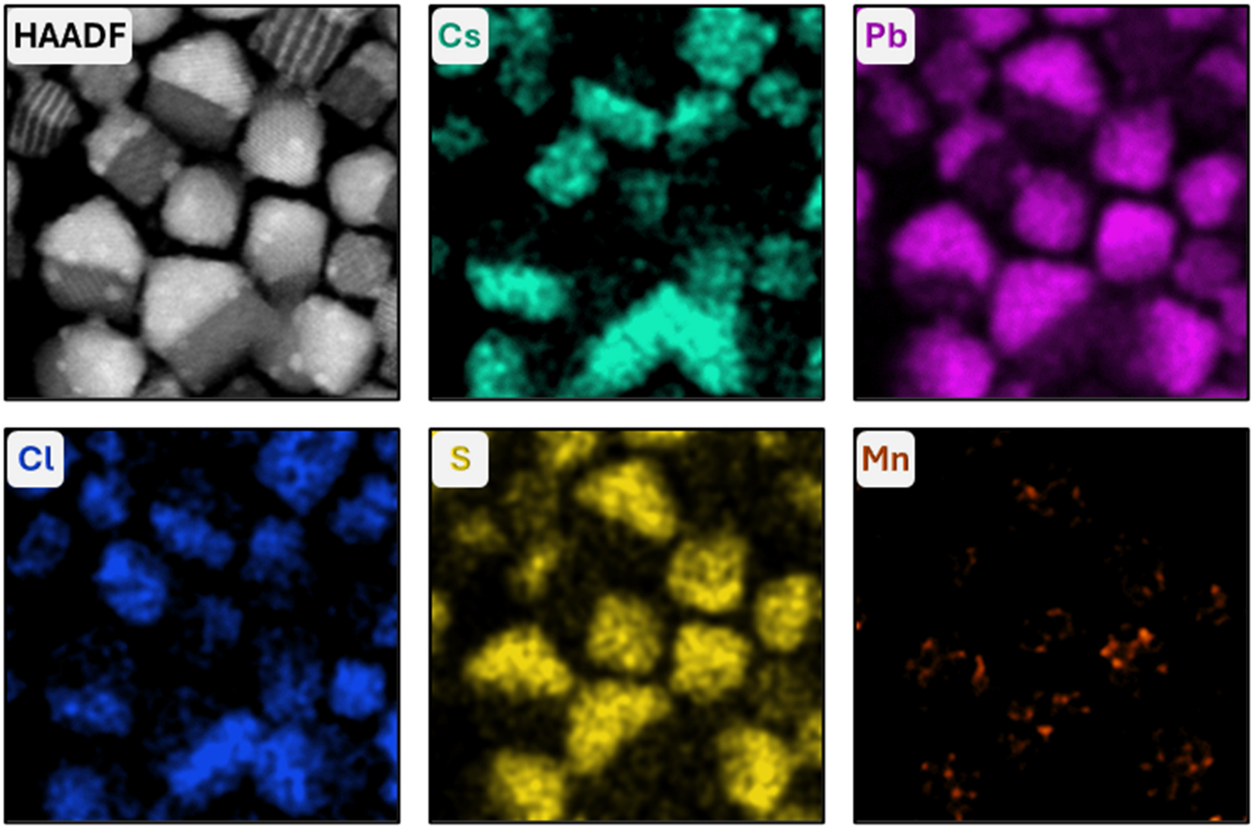

Figure S1. STEM-HAADF image of $CsPbCl_3$-PbS heterostructures (case sample "220 ^oC/S-ODE", presented in Figure 1a) with the corresponding EDX elemental maps for cesium, lead, chlorine, sulfur, and manganese indicating a small presence of manganese on the nanocrystal surface.

Table S1. Summary of the elemental analysis obtained from the EDX spectrum of **Figure S1** expressed in atomic percentage (%) and mass percentage (%).

Z	Element	Atomic (%)	Mass (%)	Fit Error (%)
16	S	21.42	5.32	3.79
17	Cl	17.18	4.72	0.73
25	Mn	1.74	0.74	1.06
55	Cs	11.43	11.77	0.05
82	Pb	48.23	77.45	0.09

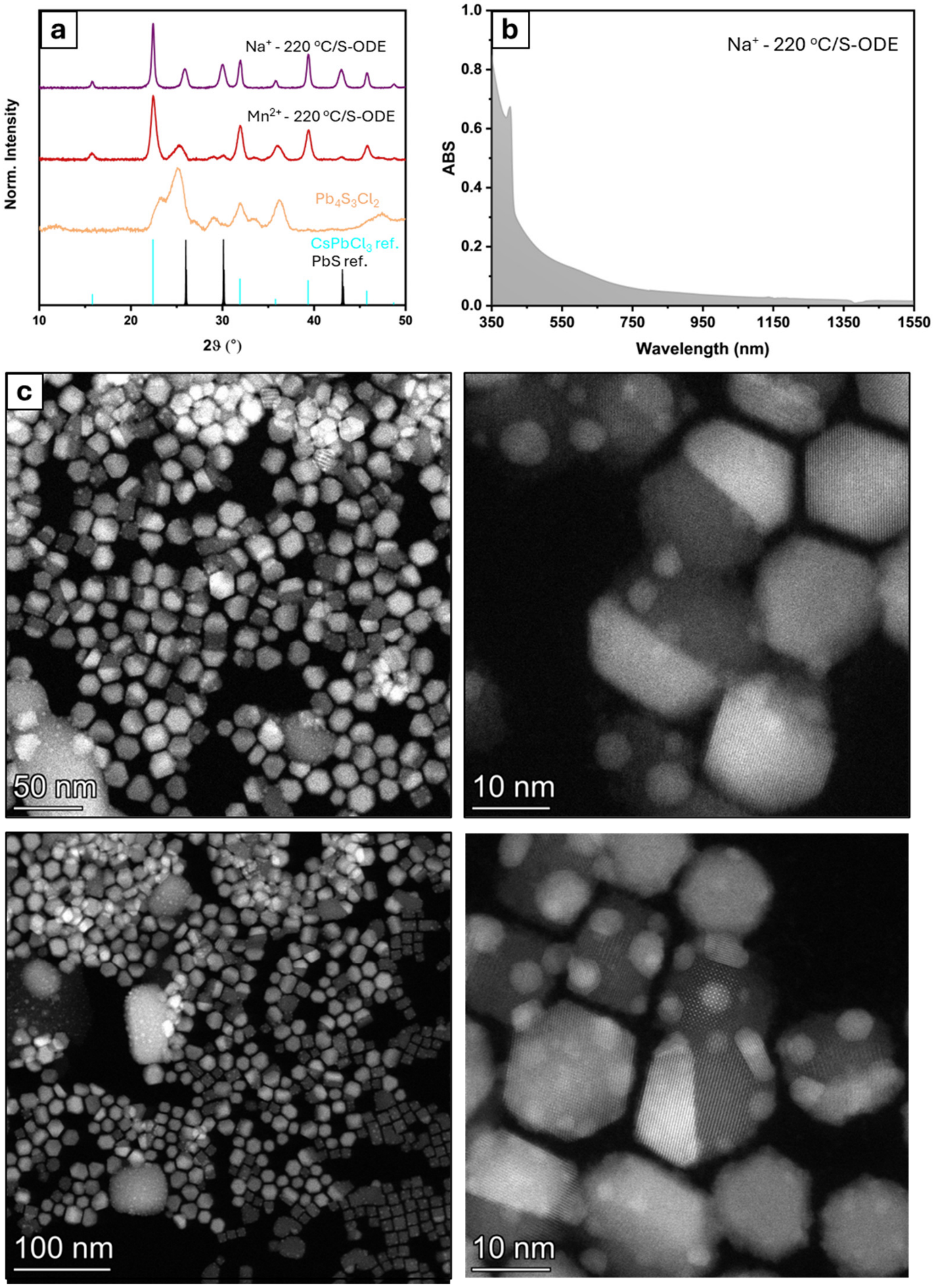

Figure S2. (a) XRD diffraction patterns of the synthesis route "220 ºC/S-ODE" in which either Na-OL (purple) or Mn-OL (red) is added. We report, for comparison, the diffraction pattern of a sample of $Pb_4S_3Cl_2$ chalcohalide nanocrystals (orange) and $CsPbCl_3$, and PbS bulk reference patterns (cyan and black, respectively). The peak at ~ 43º indicates the formation PbS. (b) Optical absorption spectrum of the sample from the synthesis "220 ºC/S-ODE" in which Na-OL has been used instead of Mn-OL. (c) STEM-HAADF images of the same sample as in (b).

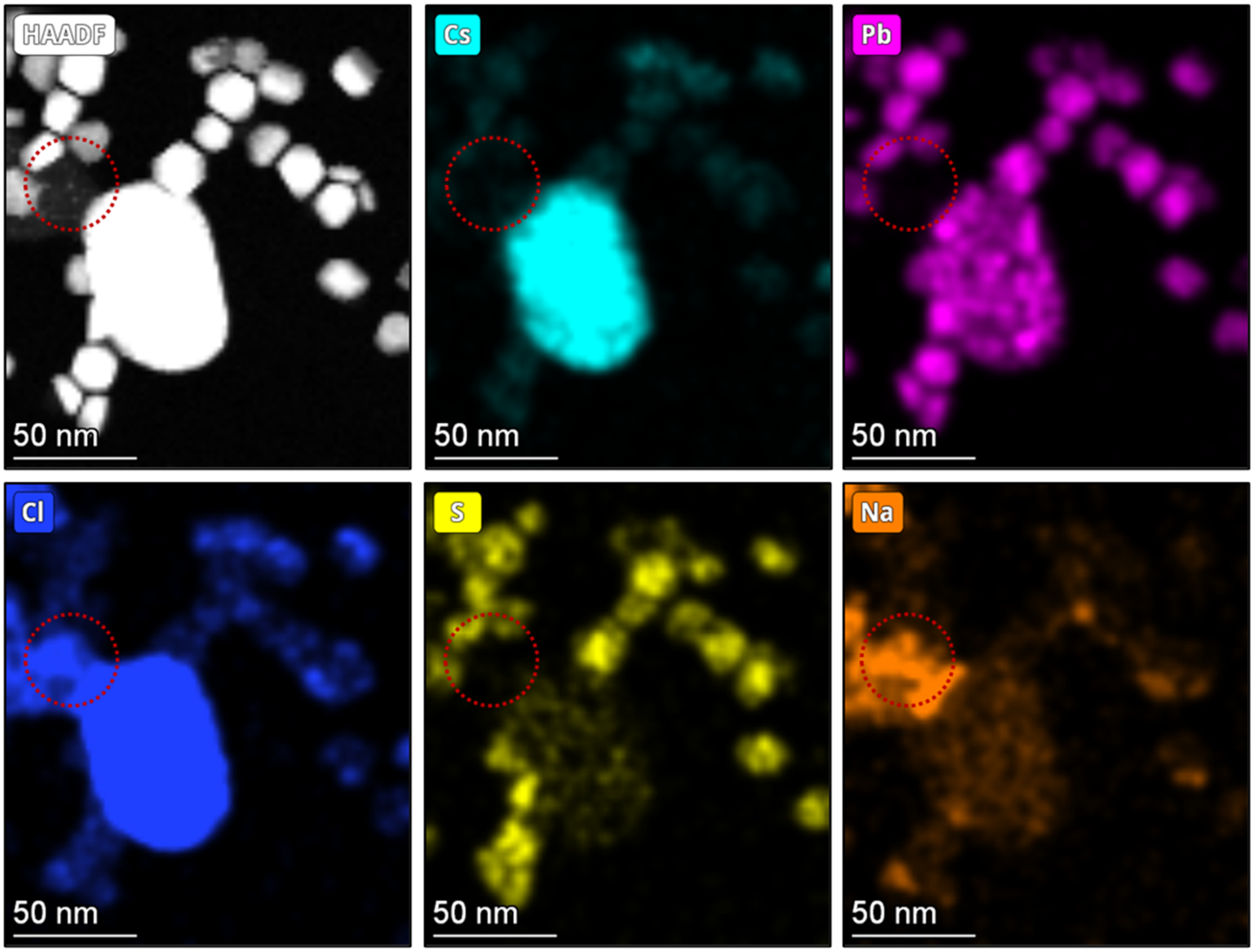

Figure S3. STEM-HAADF images of the sample case "220 ºC/S-ODE" in which Na-OL has been used instead of Mn-OL, along with the corresponding EDX elemental maps for cesium, lead, chlorine, sulfur, and sodium. The area marked with a red dashed cycle corresponds to an amorphous aggregate containing mainly Na and Cl. A large nanocrystal, containing mainly Cs, Pb, and Cl (along with traces of S and Na) is also present.

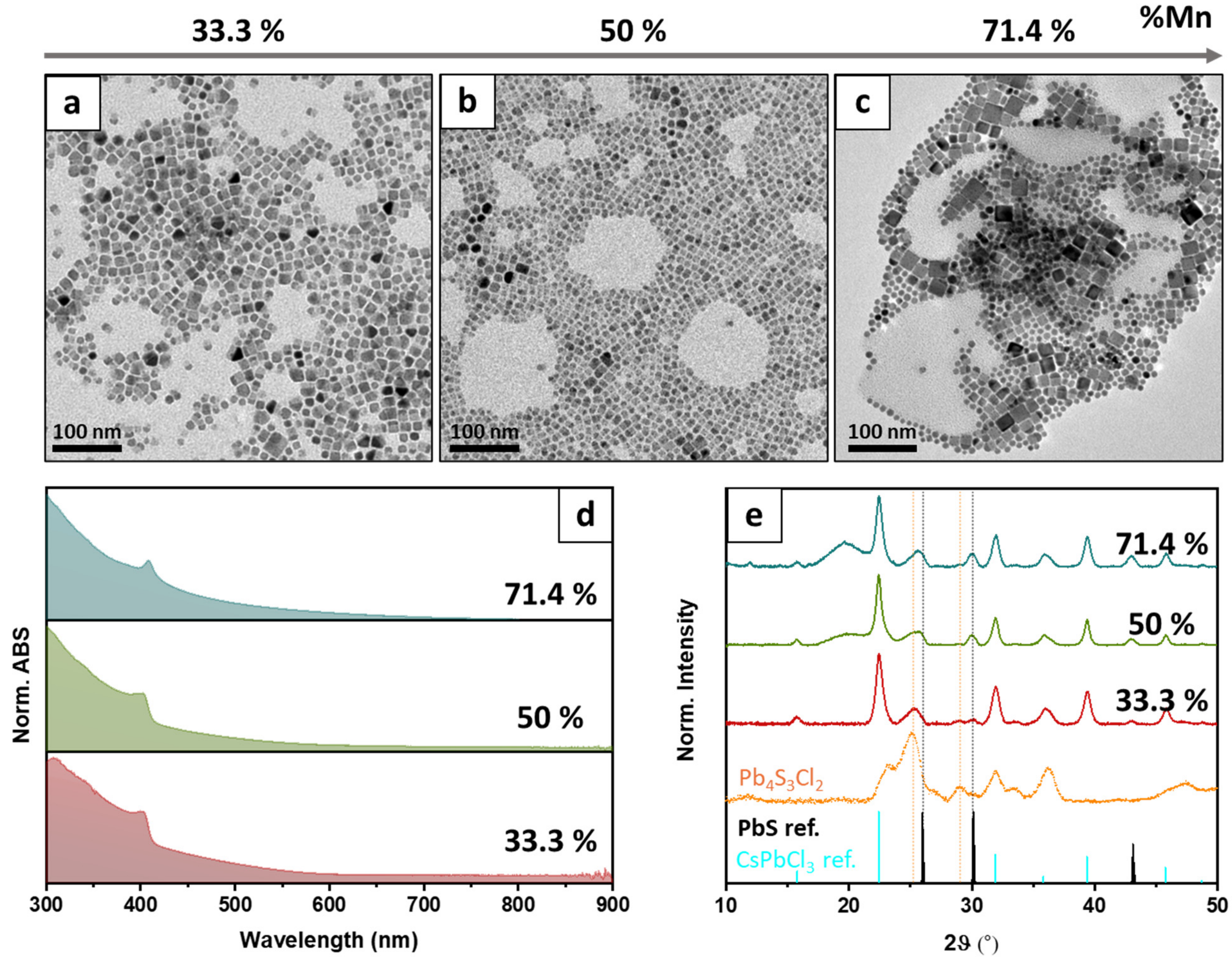

Figure S4. Influence of Mn^{2+} feed ratio in the $CsPbCl_3$-PbS heterostructures synthesis (using Sulfur-ODE as a sulfur source) by varying the concentration of Mn^{2+} added in the reaction system (X_{Mn} = [Mn]/([Mn]+[Pb])*100). (a-c) TEM images and (d) optical absorption spectra of heterostructures obtained with different Mn-oleate precursor concentrations. (e) XRD patterns of $CsPbCl_3$-PbS heterostructures obtained with different Mn^{2+} concentrations (blue, green, and red) and comparison with $Pb_4S_3Cl_2$ chalcohalides diffraction pattern (orange) and $CsPbCl_3$, and PbS reference patterns (cyan, and black respectively).

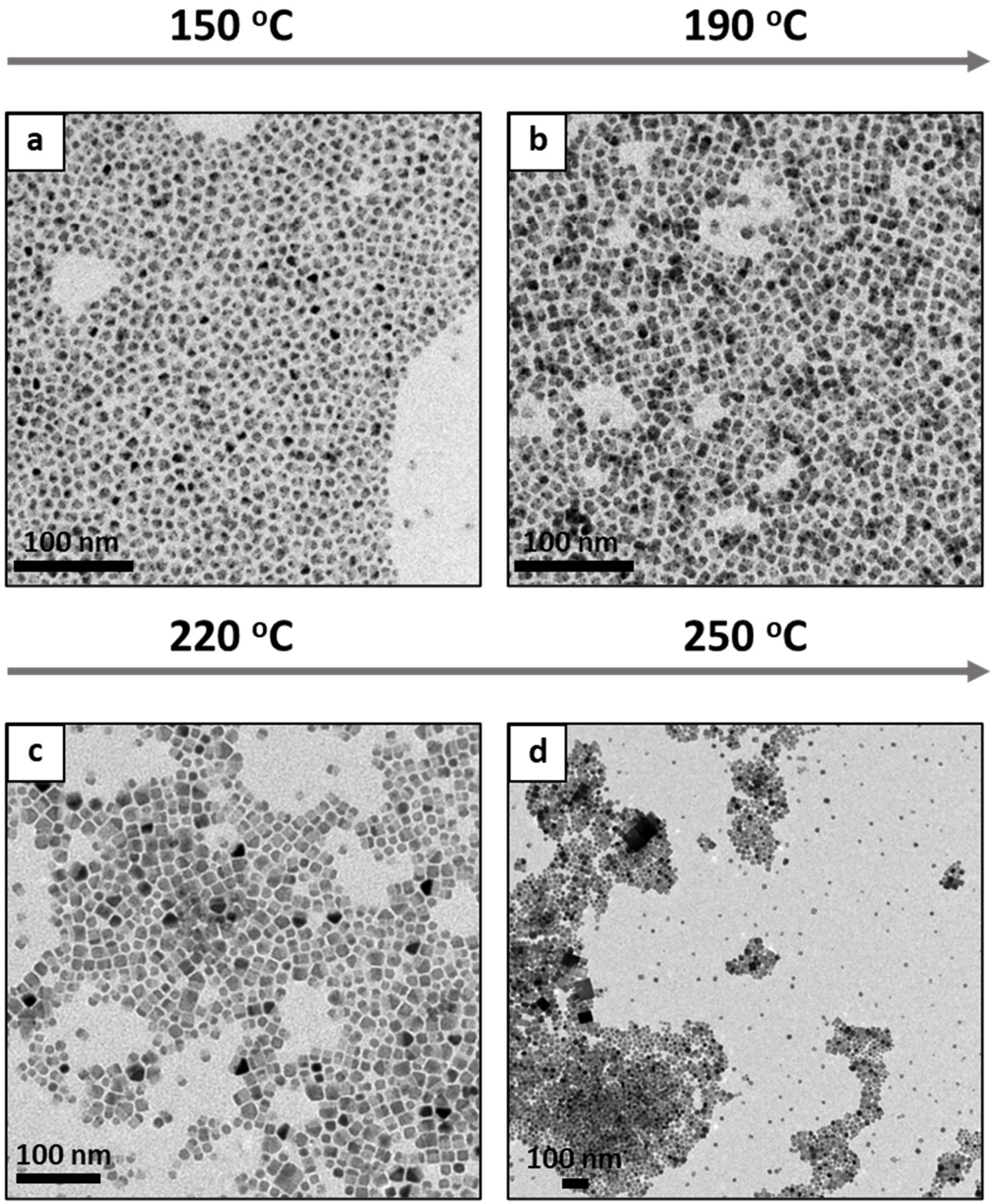

Figure S5. Influence of the reaction temperature on the formation of $CsPbCl_3$-PbS heterostructures using S-ODE as a sulfur source. TEM images of the heterostructures product were obtained at different reaction temperatures (150 to 250 °C) in the S-ODE synthesis and a reaction time of 5 min.

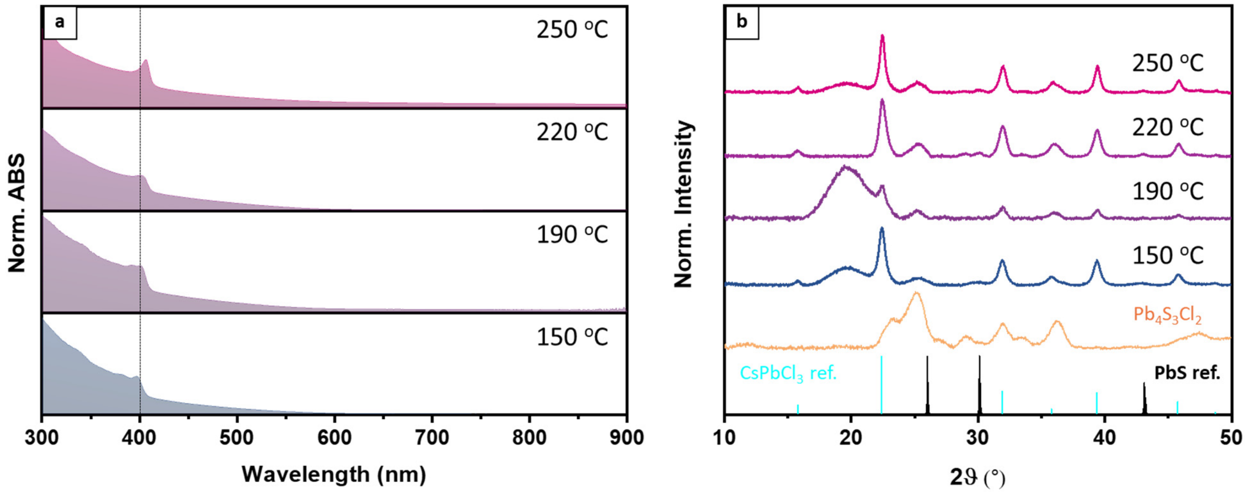

Figure S6. Influence of reaction temperature on the formation of $CsPbCl_3$-PbS heterostructures using S-ODE as a sulfur source. Reaction time is constant at 5 min. (a) UV-Vis-NIR absorption spectra of $CsPbCl_3$-based heterostructures obtained at different reaction temperatures and using S-ODE as a sulfur source. (b) XRD patterns of $CsPbCl_3$-based heterostructures obtained at different reaction temperatures and using S-ODE as a sulfur source and comparison with $Pb_4S_3Cl_2$ chalcohalides diffraction pattern (orange) and $CsPbCl_3$, and PbS reference patterns (cyan and black, respectively). The formation of the $CsPbCl_3$-PbS heterostructures population with the temperature is evidenced by the appearance of diffraction peaks at ~ 30° and 43°. The strong reflections attributed to the chalcohalide phase are representative of the dominant $CsPbCl_3$-$Pb_4S_3Cl_2$ populations observed in corresponding TEM images in Figure S2.

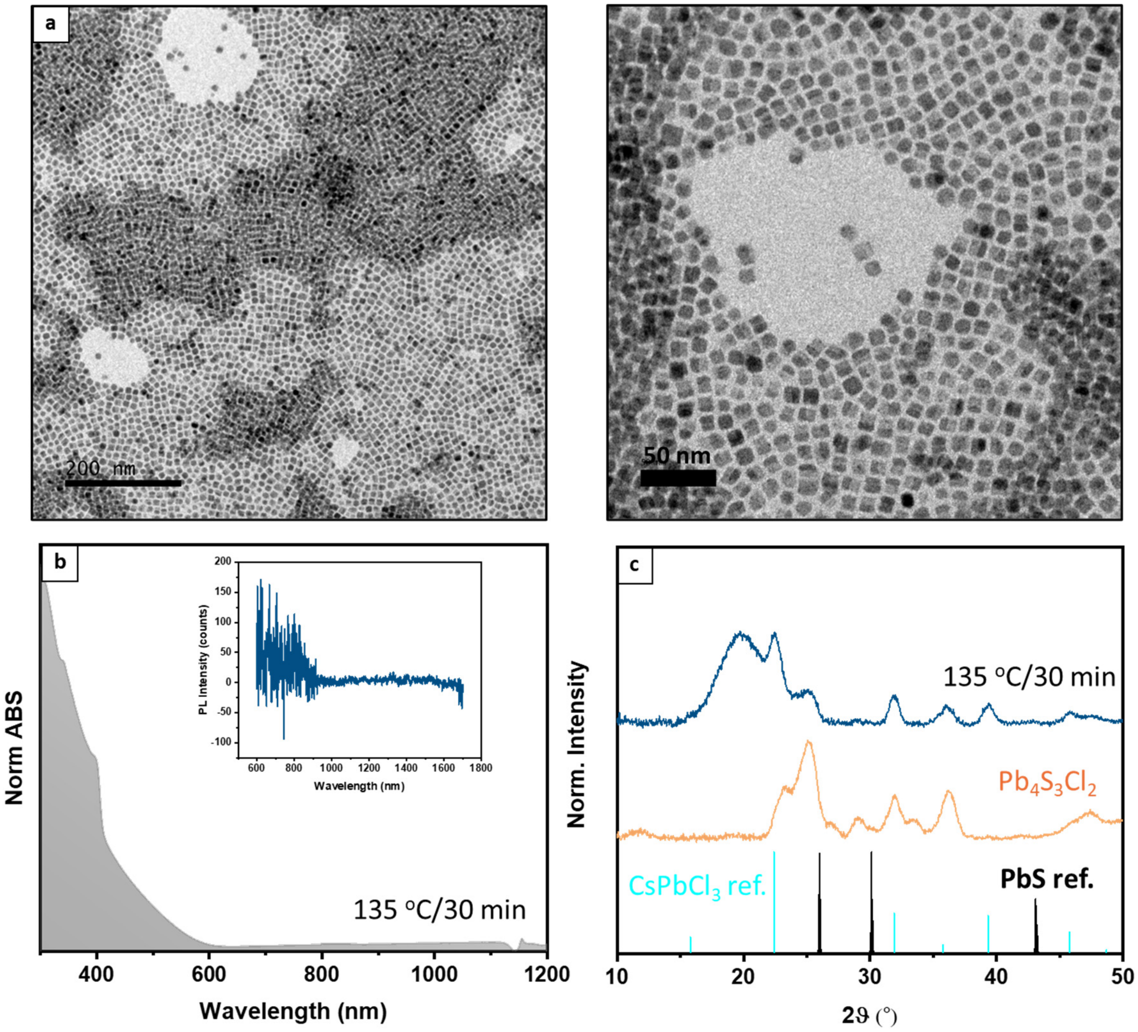

Figure S7. Influence of low reaction temperature (135 °C) on the formation of $CsPbCl_3$-PbS heterostructures using sulfur-ODE as a sulfur source. The reaction time is 30 min. (a) TEM images, (b) UV-Vis-NIR absorption and PL (inset) spectra, and (c) XRD patterns of the heterostructures product obtained at low temperatures (135 °C). XRD reference patterns are the following: orange for $Pb_4S_3Cl_2$, cyan for $CsPbCl_3$, and black for PbS.

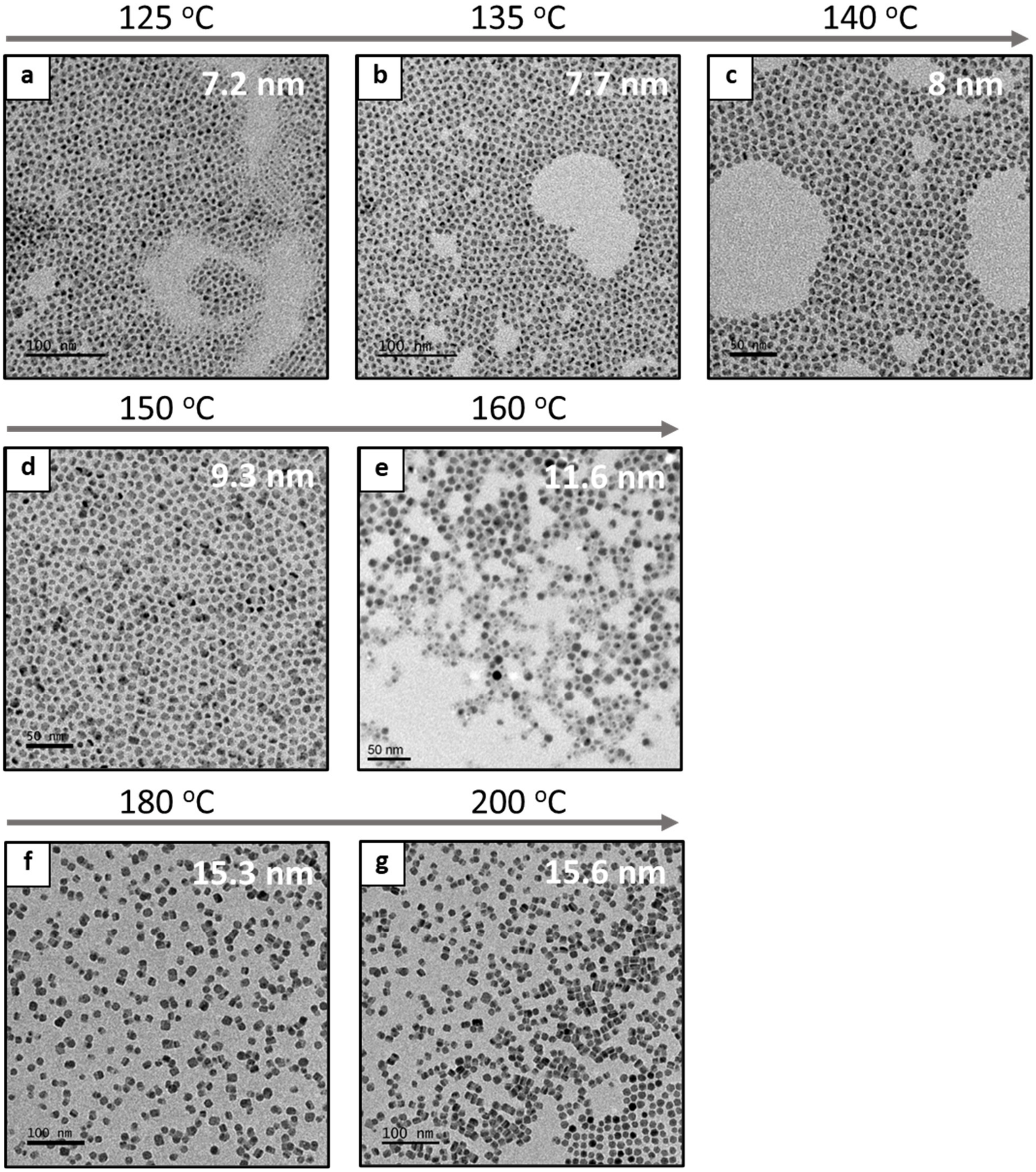

Figure S8. Influence of the reaction temperature on the formation of $CsPbCl_3$-PbS heterostructures using TMS-ODE as a sulfur source. The reaction time is 5 min. TEM images of $CsPbCl_3$-PbS heterostructures were obtained at different reaction temperatures (125 – 200 °C). The images evidenced an increase in the heterostructure size with the reaction temperature. At temperatures higher than 160 °C, the heterostructures were more heterogeneous in size and shape.

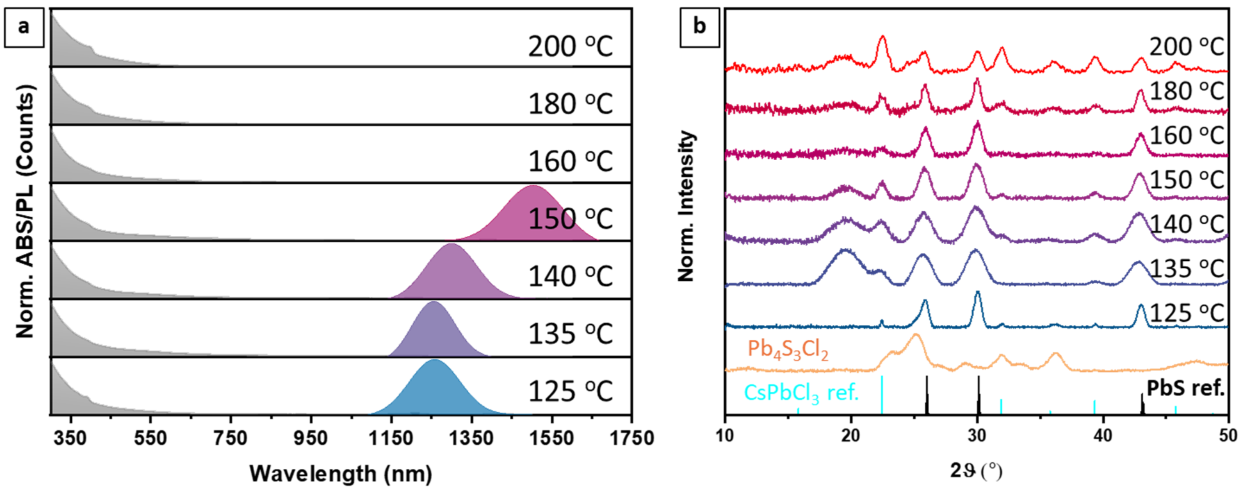

Figure S9. Influence of reaction temperature on the formation of the $CsPbCl_3$-based heterostructures using TMS-ODE as a sulfur source. Reaction time is constant at 5 min. (a) UV-Vis-NIR absorption and PL spectra, and (b) XRD patterns of $CsPbCl_3$-based heterostructures obtained at different reaction temperatures (125 – 200 °C). The XRD reference patterns (bottom) are the following: orange for $Pb_4S_3Cl_2$ chalcohalides, cyan for $CsPbCl_3$, and black for PbS. For reaction temperatures over 180 °C, the presence of $Pb_4S_3Cl_2$ chalcohalides as subproduct is evidenced by the appearance of a diffraction peak at ~ 25°.

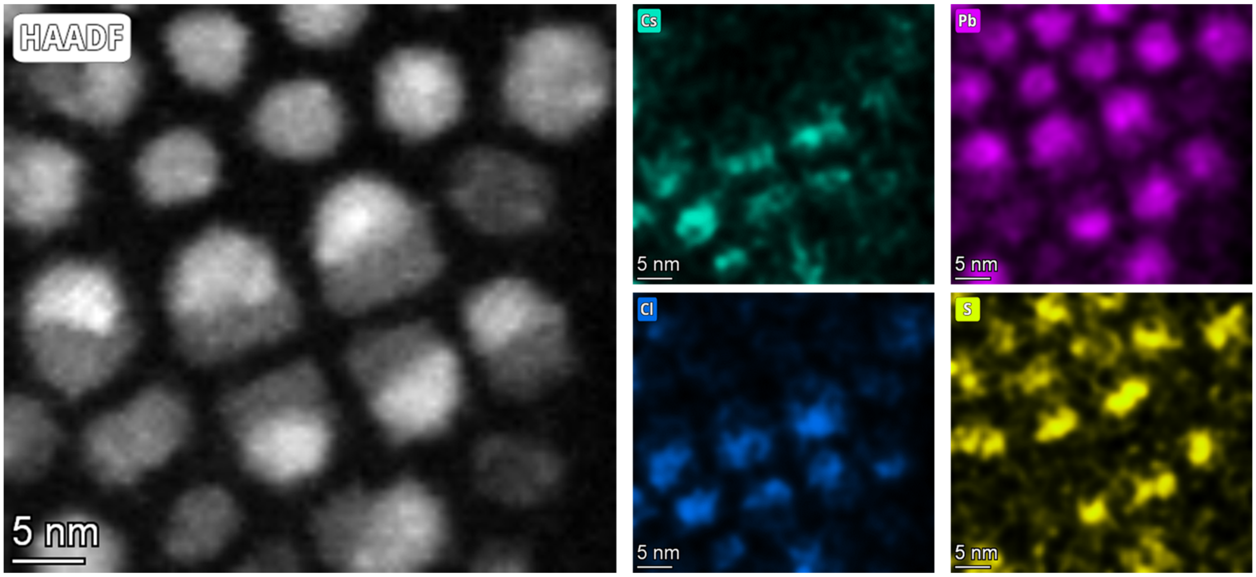

Figure S10. HAADF STEM image of $CsPbCl_3$-PbS heterostructures (case sample "125 °C/TMS", as described in Figure 1c) with the corresponding EDX elemental maps for cesium, lead, chlorine, and sulfur.

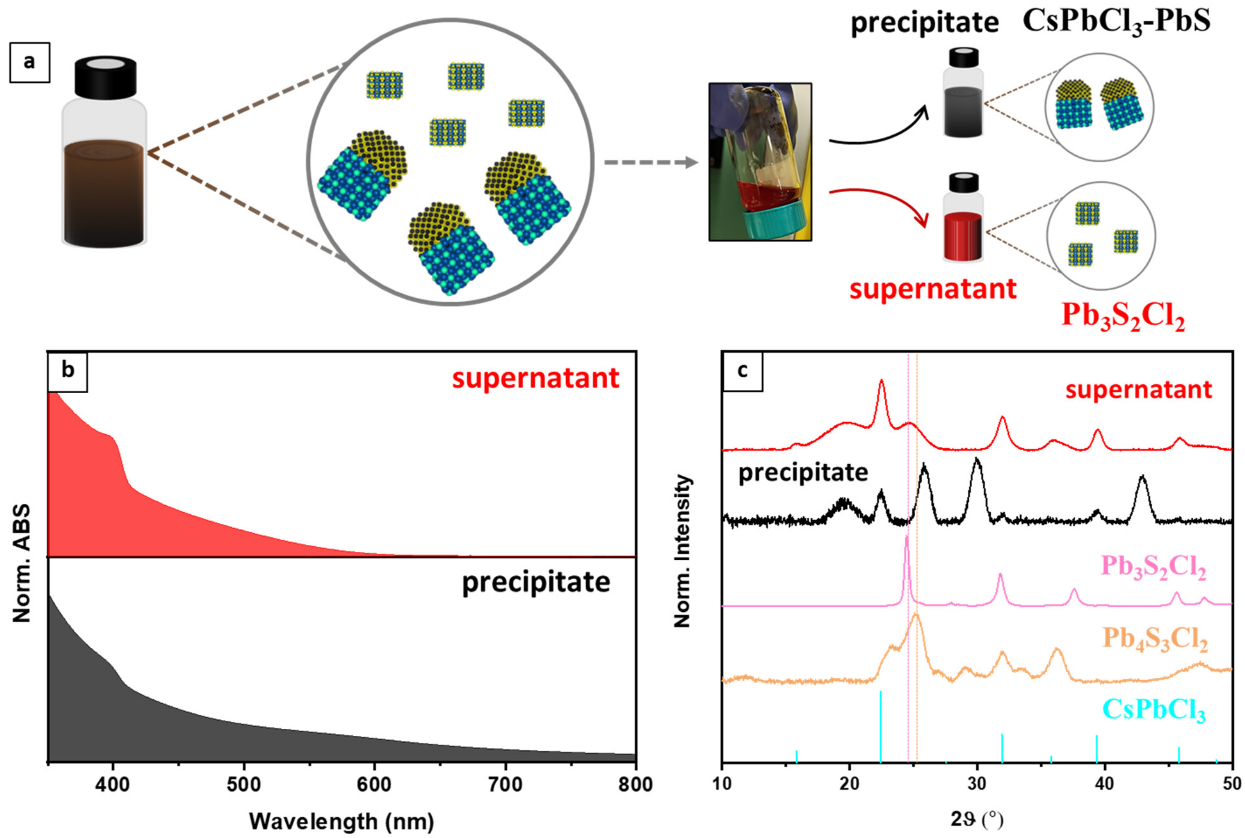

Figure S11. (a) Sketch of the purification process of a crude solution obtained at a reaction temperature of 150 ºC using TMS as the sulfur source (case sample "150 ºC/TMS", as described in Figure 1b). The 2 products obtained in the reaction ($CsPbCl_3$-PbS heterostructures and $Pb_3S_2Cl_2$ chalcohalides) are easily separated via centrifugation, remaining the $Pb_3S_2Cl_2$ chalcohalides in the supernatant (red) and precipitating the PbS based heterostructures (black). (b) UV-Vis-NIR absorption spectra of the supernatant and precipitate resulted from the purification process described in (a). The optical absorption in the 400-600 nm range indicates the presence of $Pb_3S_2Cl_2$ chalcohalides in the supernatant (top spectrum) accompanied by the absorption of $CsPbCl_3$ perovskite NCs. The absorption in the NIR range in the bottom spectrum with the excitonic peak at ~ 400 nm evidences the presence of $CsPbCl_3$-PbS heterostructures. (c) XRD patterns of the supernatant and precipitate resulted from the purification process described in (a) and comparison with $Pb_3S_2Cl_2$ chalcohalides diffraction pattern (pink), $Pb_4S_3Cl_2$ chalcohalides diffraction pattern (orange) and $CsPbCl_3$ reference pattern (cyan). The diffraction peak at ~ 24º indicates the presence of the $Pb_3S_2Cl_2$ phase in the supernatant (red). The absence of ~ 24º diffraction peak in the precipitate (black) confirms the $CsPbCl_3$-PbS phase purity.

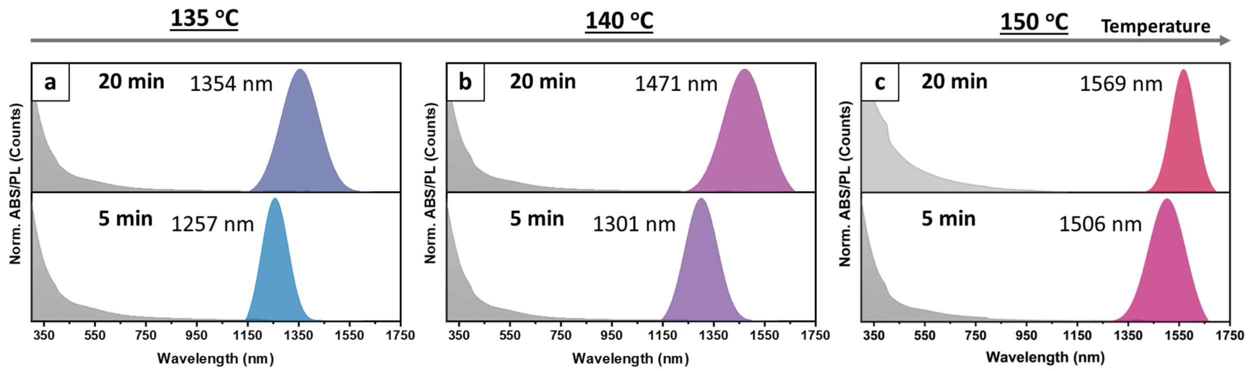

Figure S12. Influence of reaction temperature and reaction time on the growth of the $CsPbCl_3$-PbS heterostructures using TMS as a sulfur source. (a-c) UV-Vis-NIR absorption and PL spectra of $CsPbCl_3$-PbS heterostructures obtained at different reaction temperatures: 135 ºC (a), 140 ºC (b), and 150 ºC (c); and different reaction times: 5 min (bottom spectra), and 20 min (top spectra).

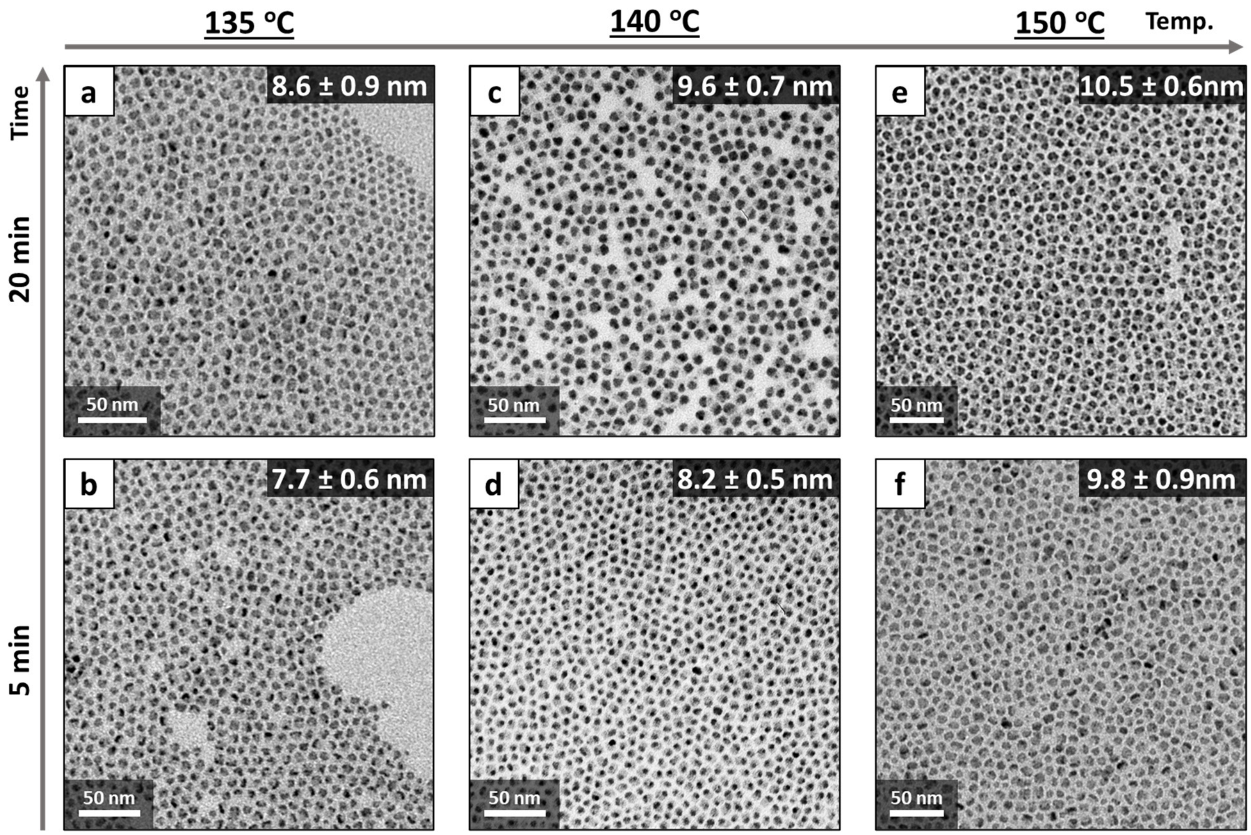

Figure S13. Influence of reaction temperature and reaction time on the growth of the $CsPbCl_3$-PbS heterostructures using TMS as a sulfur source. (a-f) TEM images of $CsPbCl_3$-PbS heterostructures obtained at different reaction temperatures: 135 °C (a,b), 140 °C (c,d), and 150 °C (e,f); and different reaction times: 5 min (bottom images), and 20 min (top images).

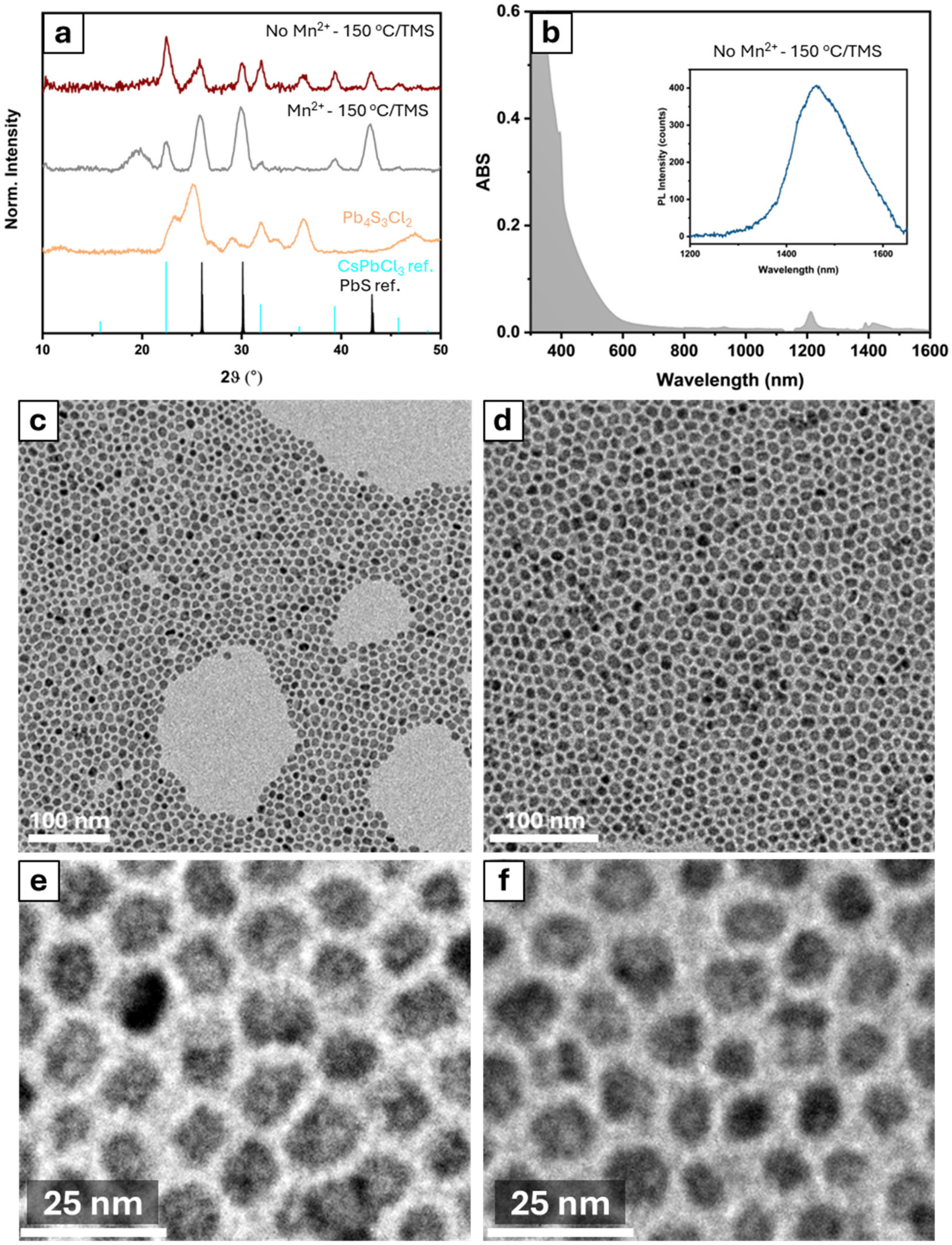

Figure S14. Analysis of the synthesis of case sample "150 °C/TMS" in the absence of Mn^{2+}. (a) XRD diffraction patterns of the products synthesized via the "150 °C/TMS" method with (grey) and without (red) Mn-OL, and comparison with the $Pb_4S_3Cl_2$ chalcohalides diffraction pattern (orange) and $CsPbCl_3$, and PbS reference patterns (cyan, and black, respectively). The absence of Mn^{2+} in the reactions yields a product containing both $Pb_4S_3Cl_2$ and PbS phases (along with the perovskite) as indicated by the peaks at 25.2° and 26.2°, respectively. (b) Optical absorption and PL spectra of the sample synthesized with "150 °C/TMS" in the absence of Mn-OL. (c,d) TEM images of the same sample demonstrate a mixture of productcs in addition to the $CsPbCl_3$-PbS heterostructures. Therefore, it is likely that the trimethylsilyl group of TMS can act as an effective Cl^- scavenger. (e,f) Enlarged areas of the TEM image presented in (c).

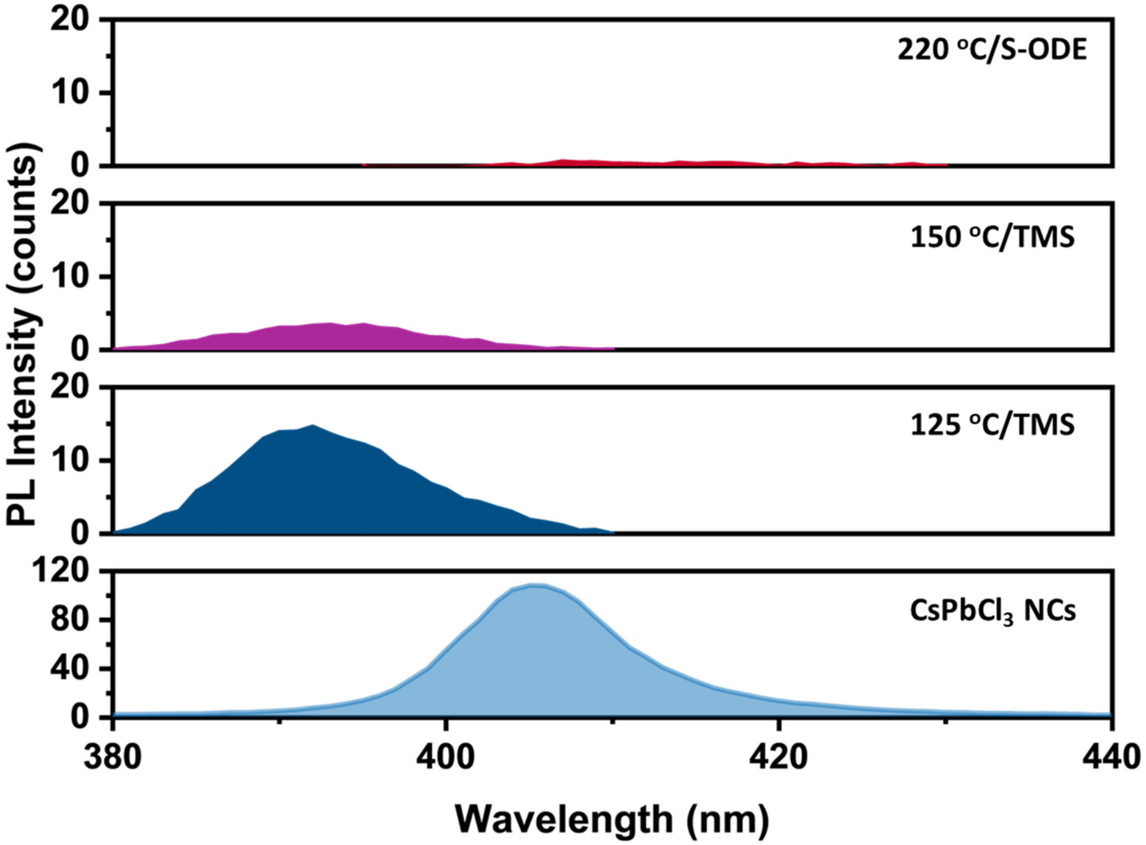

Figure S15. Photoluminescence spectra in the visible range capturing the emission originated from the perovskite domain (λexc=350 nm). The four spectra correspond to pure $CsPbCl_3$ NCs and the three cases/samples of heterostructures represented in Figure 1. Notably, the $CsPbCl_3$ NCs were synthesized based on the clusters-based approach used for the synthesis of the heterostructure. This suggests similar surface capping (passivation) between the pure perovskite NCs and heterostructures. In every case, the perovskite emissions are severely quenched when they are bound to the PbS or $Pb_4S_3Cl_2$ domains.

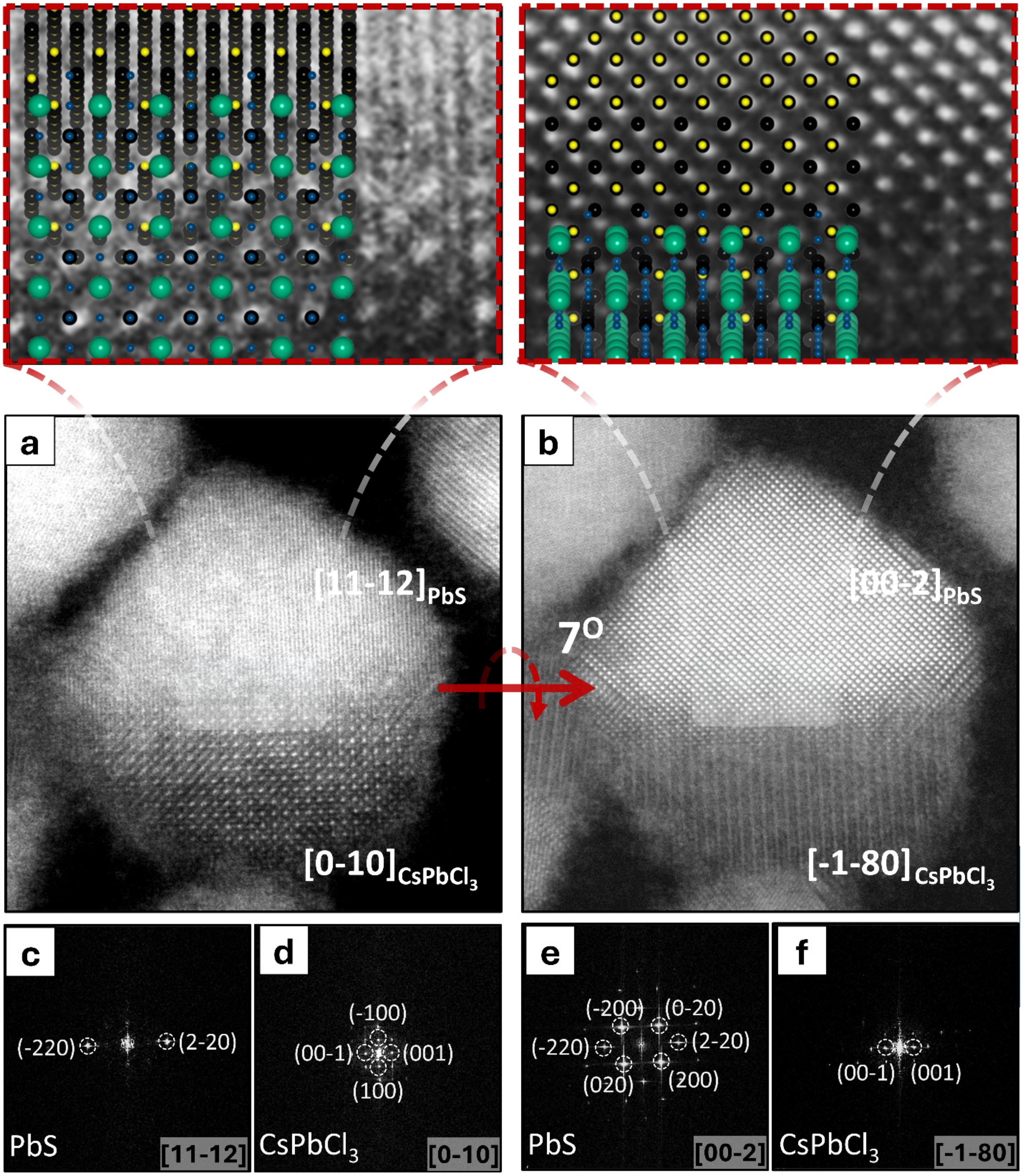

Figure S16. **Structural and compositional analysis of $CsPbCl_3$-PbS epitaxial heterostructures.** (a-b) HAADF STEM images of the same heterostructure tilted by 7° around the x-axis to align both domains on low-index zone axes. (c,d), and (e,f) represent FFT patterns of the image a, and b, respectively. For each image, we present two FFTs corresponding to the two distinct domains of the heterostructure. (a,b upper panels) represent magnified areas of the STEM images. The constructed 3D model fits the atomic columns in this projection as well, validating the model. FFT patterns (c,d, or e,f) suggest that the zone axes of the heterostructure projection presented in Figure 2b are parallel planes in this orthogonal heterostructure projection presented here (a or b).

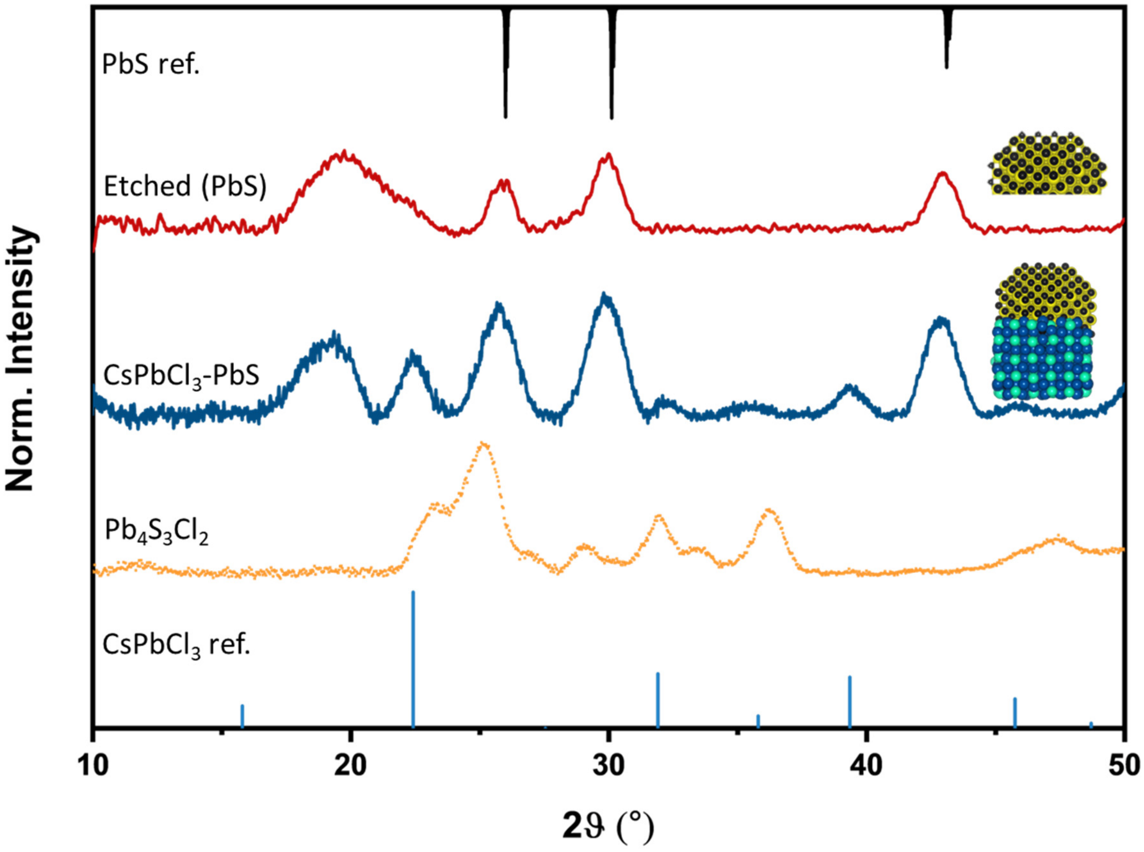

Figure S17. XRD patterns of $CsPbCl_3$-PbS heterostructures (sample "150 °C/TMS" corresponding to the blue pattern), and the etched $CsPbCl_3$-PbS heterostructures (thus, PbS NCs represented in the red pattern). Additional XRD patterns are displayed for comparison: Experimental pattern of $Pb_4S_3Cl_2$ (orange), and reference patterns of $CsPbCl_3$ (cyan lines), and PbS (black lines). The absence of reflections at 22.6°, 32°, and 39.5° in the etched sample, confirms the successful etching of the perovskite domain, and thus obtaining pure PbS NCs. The broad peak at approximately 19° is attributed to the fumed silica used for performing the XRD measurements.

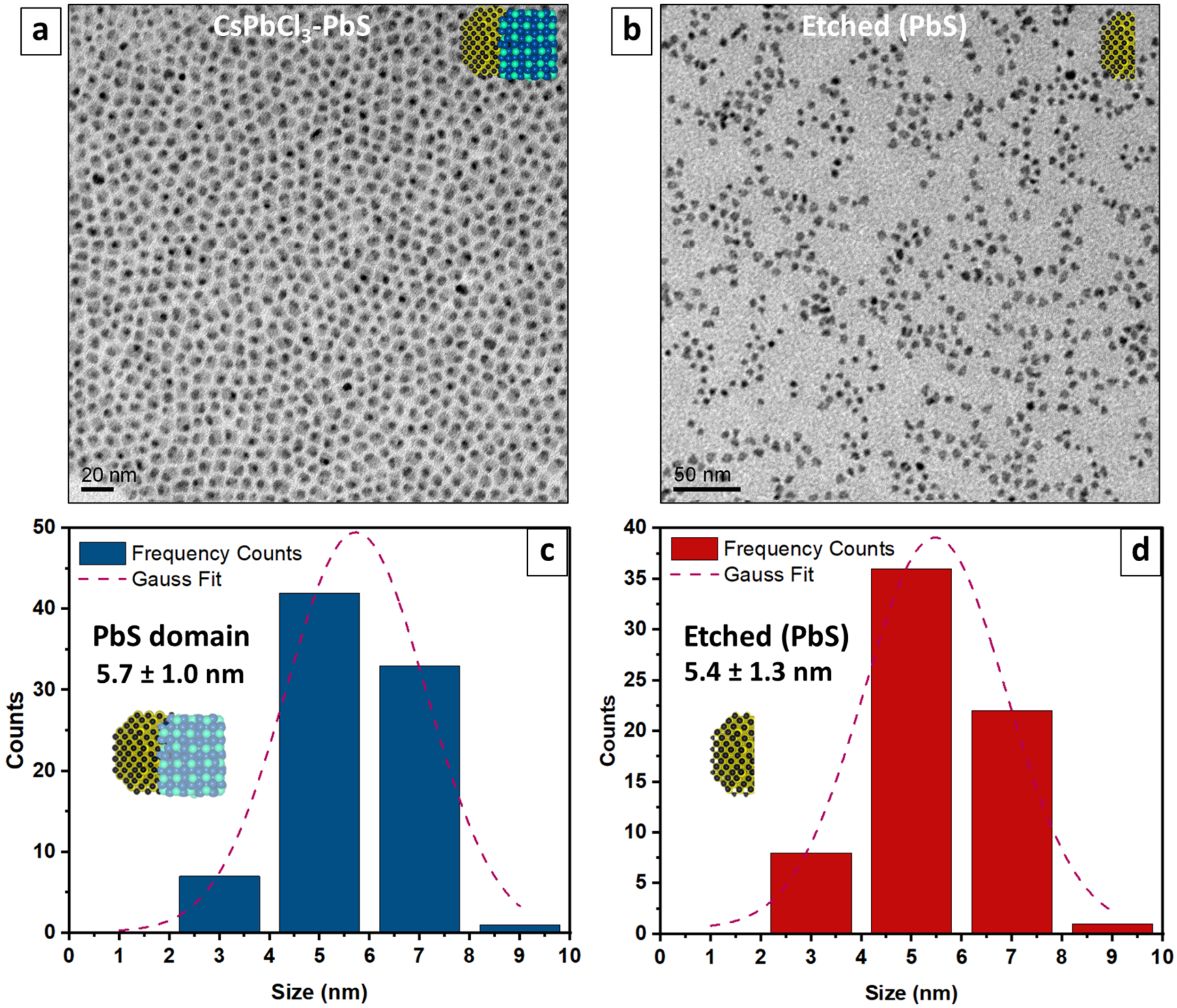

Figure S18. Morphological comparison of $CsPbCl_3$-PbS heterostructures (sample "150 ºC/TMS"), and the etched $CsPbCl_3$-PbS heterostructures (thus, PbS NCs). (a,b) Low magnification TEM images of $CsPbCl_3$-PbS heterostructures (panel a), and the etched $CsPbCl_3$-PbS heterostructures (i.e. PbS NCs, panel b). TEM images ensure the successful etching of the perovskite domain and thus, obtain pure PbS NCs, as already indicated by the XRD patterns (Figure S13) and optical absorption spectra (Figure 3d). (c,d) Size distribution histograms of the PbS domain in the case of the heterostructures (panel c), and after etching (panel d) comparing the size difference of the PbS before and after the etching. A slightly smaller size distribution of the PbS domain is revealed after etching. However, this difference is within the margin of error. The NC size distribution histograms were estimated using Ilastik software.

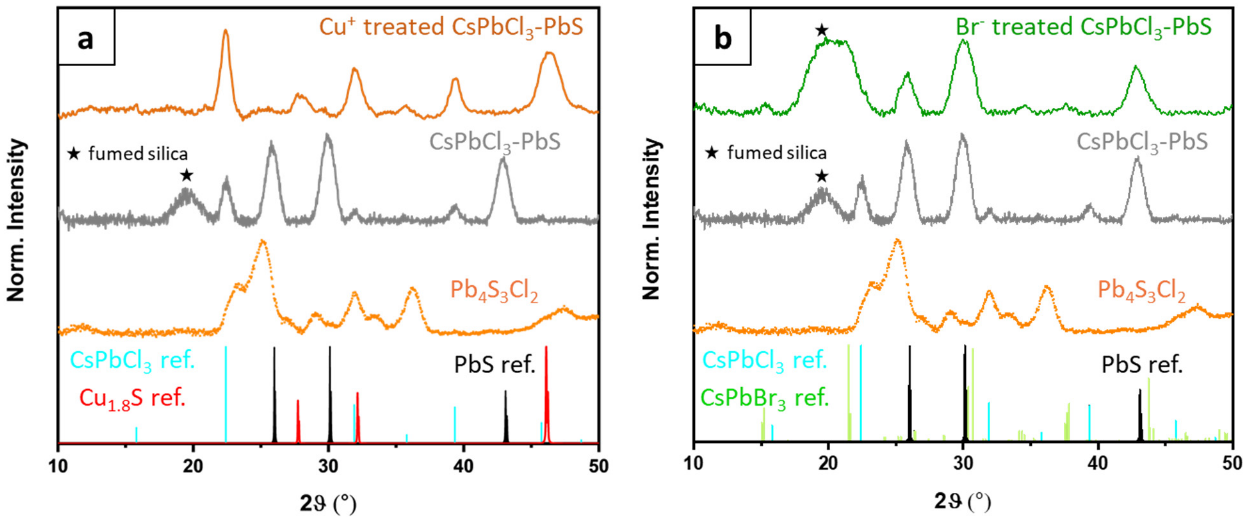

Figure S19. XRD patterns of cation (Pb^{2+}→Cu^{+}) exchanged $CsPbCl_3$-$Cu_{2-x}S$ (a), and anion (Cl^-→Br^-) exchanged $CsPbBr_3$-PbS heterostructures (b). The parent sample of $CsPbCl_3$-PbS heterostructures, used for these exchanges was defined in the text as "150 °C/TMS", with the corresponding XRD pattern presented in both panels for comparisons (grey color). (a) The Cu^+ treated $CsPbCl_3$-PbS heterostructures reveal full cation exchange to $CsPbCl_3$-$Cu_{2-x}S$ heterostructures. This is confirmed by the absence of PbS reflection peaks in the exchanged sample (at 30.2°, and 43.1°). The sub-stoichiometric $Cu_{2-x}S$ phase is ascribed to the composition of the digenite ($Fm\bar{3}m$) $Cu_{1.8}S$ phase (reference pattern represented with red lines) based on the position of the reflection peaks. The broad peak (asterisk) at approximately 19° is attributed to the fumed silica used for performing XRD measurements. Additional XRD patterns are demonstrated for comparison: Experimental pattern of $Pb_4S_3Cl_2$ (orange), and reference patterns of $CsPbCl_3$ (cyan lines), and PbS (black lines). (b) The Br^- treated $CsPbCl_3$-PbS heterostructures reveal full anion exchange to $CsPbBr_3$-PbS heterostructures. Notably, the dominant peak of the $CsPbBr_3$ orthorhombic phase overlaps with the fumed silica (£21.5°). The reference pattern of $CsPbBr_3$ (green lines) is also shown.

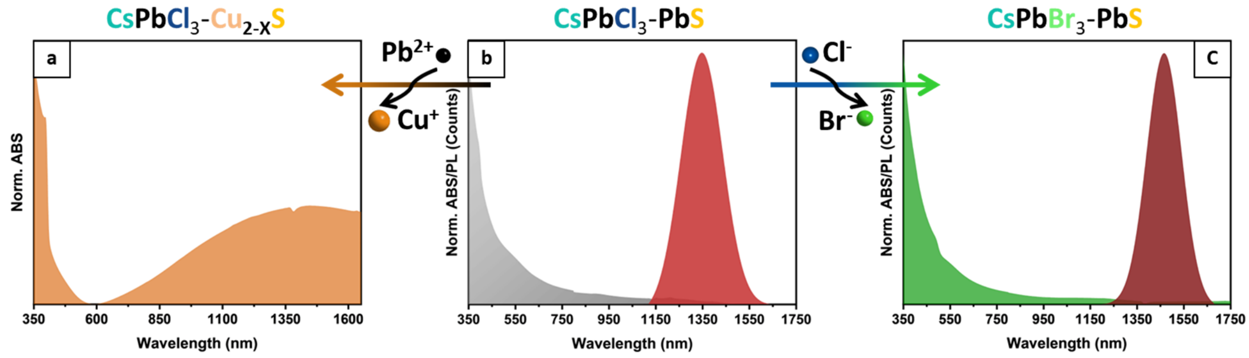

Figure S20. Optical absorption spectra of pure $CsPbCl_3$-PbS (center), cation (Pb^{2+}→Cu^{+}) exchanged $CsPbCl_3$-$Cu_{2-x}S$ (left), and anion (Cl^-→Br^-) exchanged $CsPbBr_3$-PbS heterostructures (right). The parent sample of $CsPbCl_3$-PbS heterostructures, used for these exchanges was defined in the text as "150 °C/TMS". These are the same samples that are described in Figure S15.

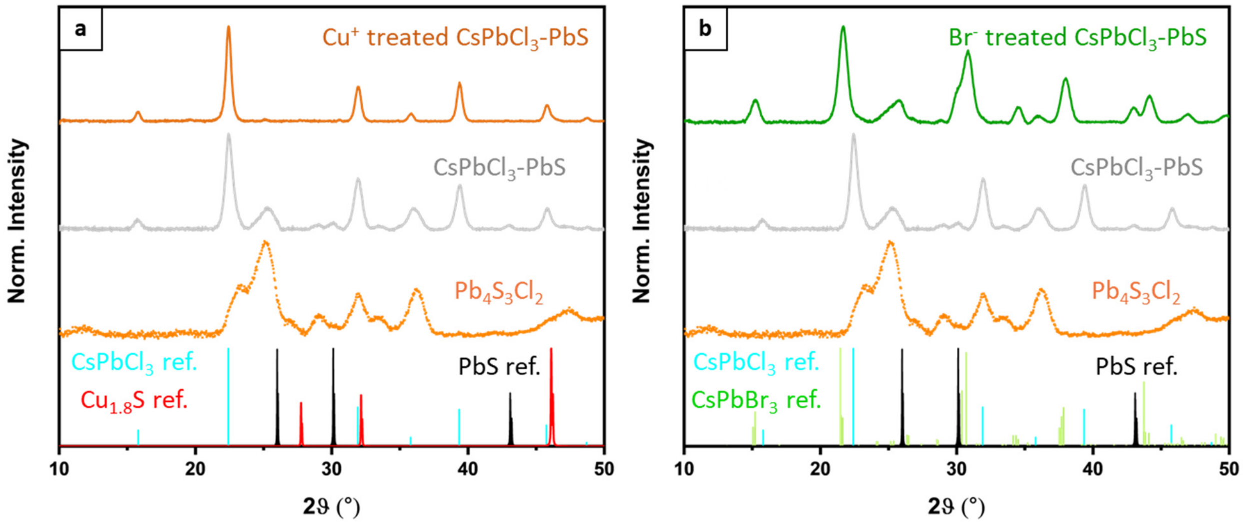

Figure S21. XRD patterns of cation (Pb^{2+}→Cu^+) exchanged $CsPbCl_3$-$Cu_{2-x}S$ (a), and anion (Cl^-→Br^-) exchanged $CsPbBr_3$-PbS heterostructures (b). The parent $CsPbCl_3$-PbS sample, used for these exchanges, was defined in the text as "220 ºC/S-ODE". The XRD pattern of the parent sample is presented in both panels for comparison (grey color).

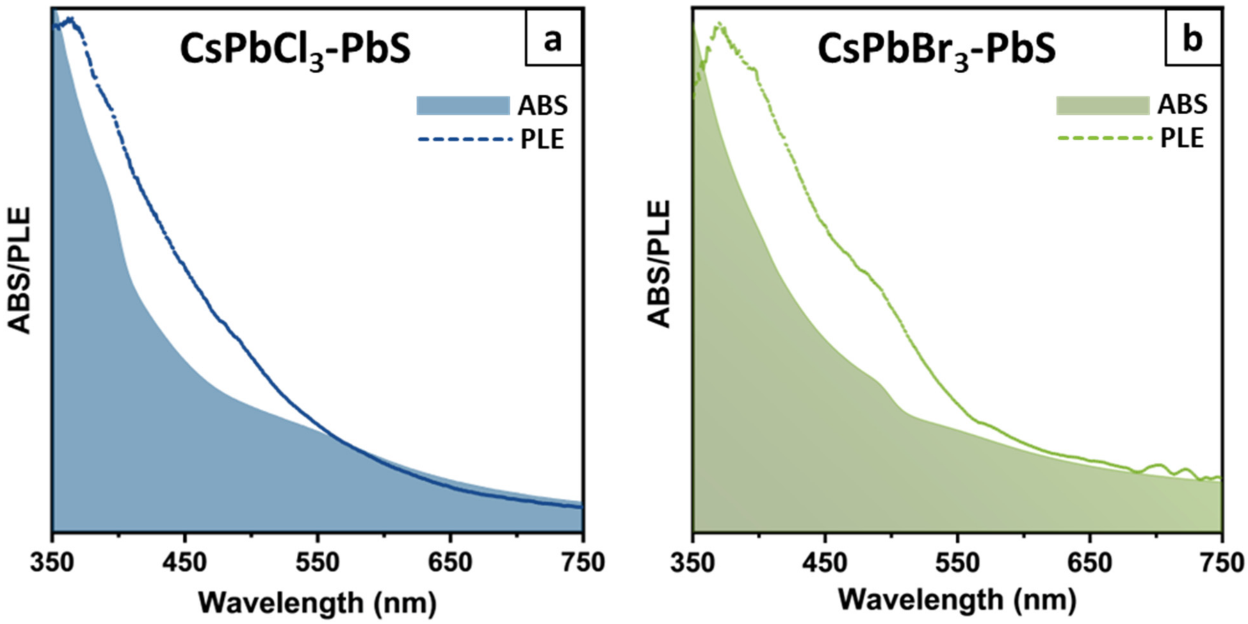

Figure S22. Optical absorption (colored curves) and PL excitation (dashed lines) spectra for both (a) $CsPbCl_3$-PbS, and anion exchanged (b) $CsPbBr_3$-PbS heterostructures (sample "150 ºC/TMS"). PLE spectrum of the $CsPbBr_3$-PbS (emission at 1470 nm) closely resembles the corresponding absorption spectrum. The peak at ~ 500 nm, which relates to the perovskite feature is more pronounced than in the case (a) of $CsPbCl_3$-PbS heterostructures. This difference could be due to the reduced contribution from absorption by PbS at the $CsPbBr_3$ exciton peak. This observation is in agreement with the Type-I alignment suggesting that photoexcited carriers in the $CsPbBr_3$ domain are transferred to the PbS domain.

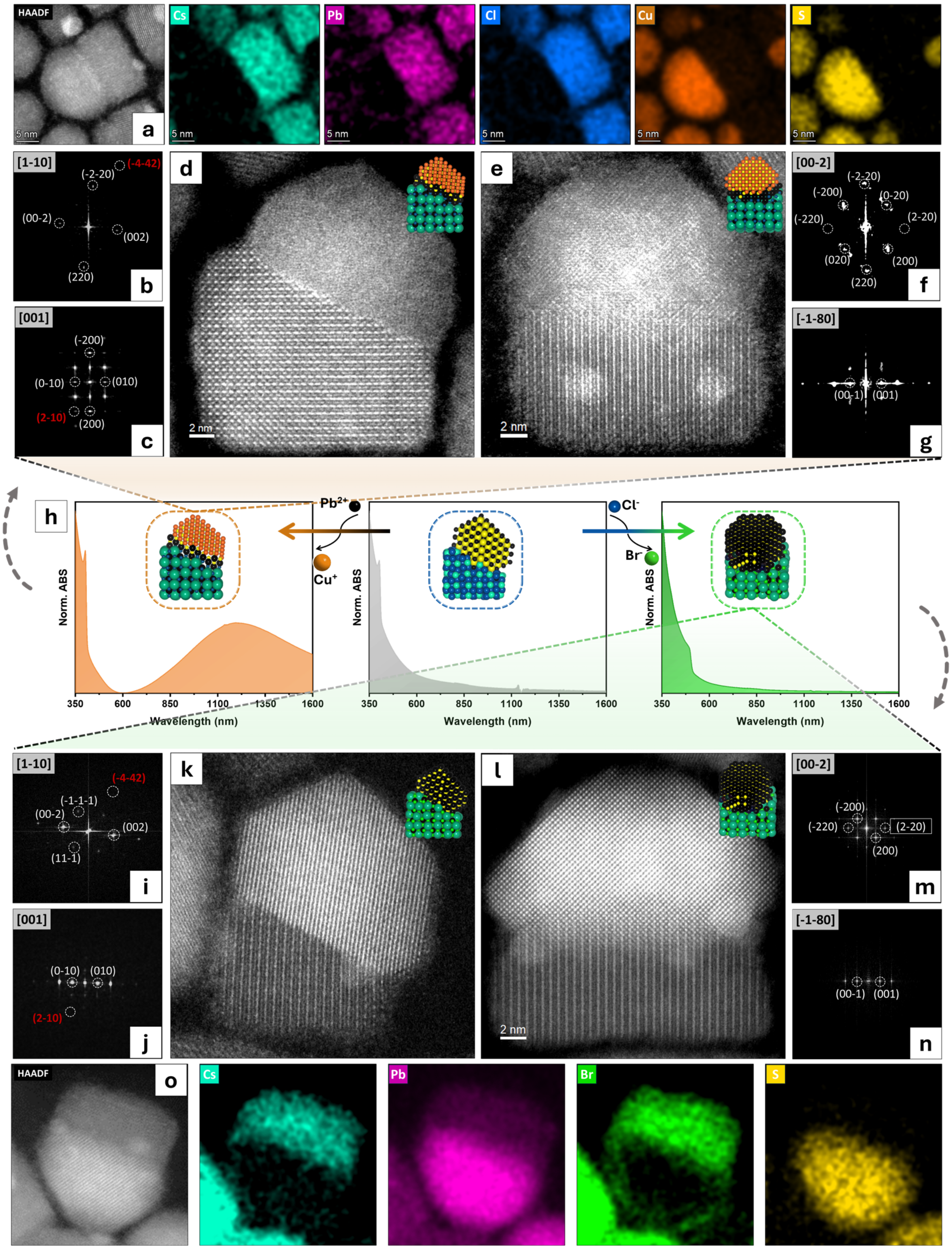

Figure S23. Phase selective cation (Pb^{2+}→Cu^{+}) and anion (Cl^{-}→Br^{-}) exchanges on $CsPbCl_3$-PbS heterostructures. (a) HAADF STEM image of a single $CsPbCl_3$-$Cu_{2-x}S$ heterostructure with the corresponding STEM-EDX elemental maps. (d,e) HAADF HR STEM images of two different cation-exchanged $CsPbCl_3$-$Cu_{2-x}S$ heterostructures in common orthogonal projections. (b,c) FFT patterns of $Cu_{2-x}S$ (b) and $CsPbCl_3$ (c) domains of the HAADF STEM image are depicted in panel d. Red dashed circles in the FFTs in panels (b) and (c) represent planes that are parallel to the interface of the heterostructure, as described in Figure 2 for the parent $CsPbCl_3$-PbS heterostructures. (f,g) FFT patterns of $Cu_{2-x}S$ (f) and $CsPbCl_3$ (g) domains of the HAADF STEM image in panel (e). FFT patterns demonstrate that the zone axes of each domain of the first projection (d) are parallel planes in the second

projection (e), ensuring orthogonality (see blue dashed spots in panels f and g). (h) Optical absorption spectra of pure $CsPbCl_3$-PbS (center), cation (Pb^{2+}→Cu^{+}) exchanged $CsPbCl_3$-$Cu_{2-x}S$ (left), and anion (Cl^-→Br^-) exchanged $CsPbBr_3$-PbS heterostructures (right). (k-l) HAADF HR STEM images of two different anion-exchanged $CsPbBr_3$-PbS heterostructures in orthogonal projections. (i,j) FFT patterns of PbS (i) and $CsPbBr_3$ (j) domains of the HAADF STEM image depicted in panel k. Red dashed circles in the FFTs represent planes that are parallel constituting the interface of heterostructure. (m,n) FFT patterns of PbS (m) and $CsPbBr_3$ (n) domains of the HAADF STEM image in panel l. (o) HAADF STEM image of a single $CsPbBr_3$-PbS heterostructure with the corresponding STEM-EDX elemental maps.

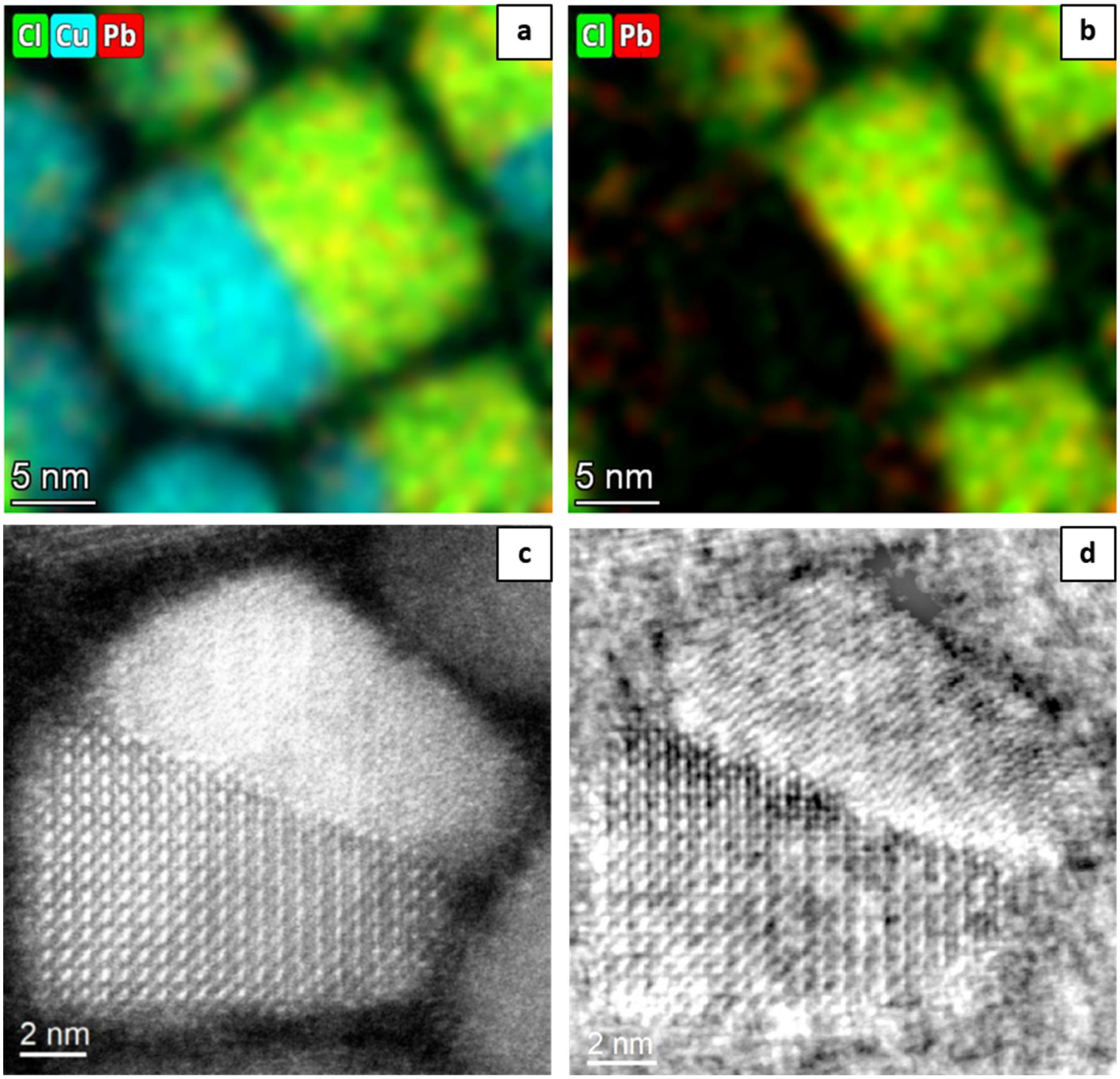

Figure S24. (a,b) STEM-EDX elemental maps of a single $CsPbCl_3$-$Cu_{2-x}S$ heterostructure, indicating a non-exchanged monolayer of PbS at the interface (where Cl^- ions are not detected, indicating that this Pb layer does not belong to the perovskite domain). (c) HAADF HR STEM- image of cation exchanged $CsPbCl_3$-$Cu_{2-x}S$. (d) iDPC STEM image of the same area.

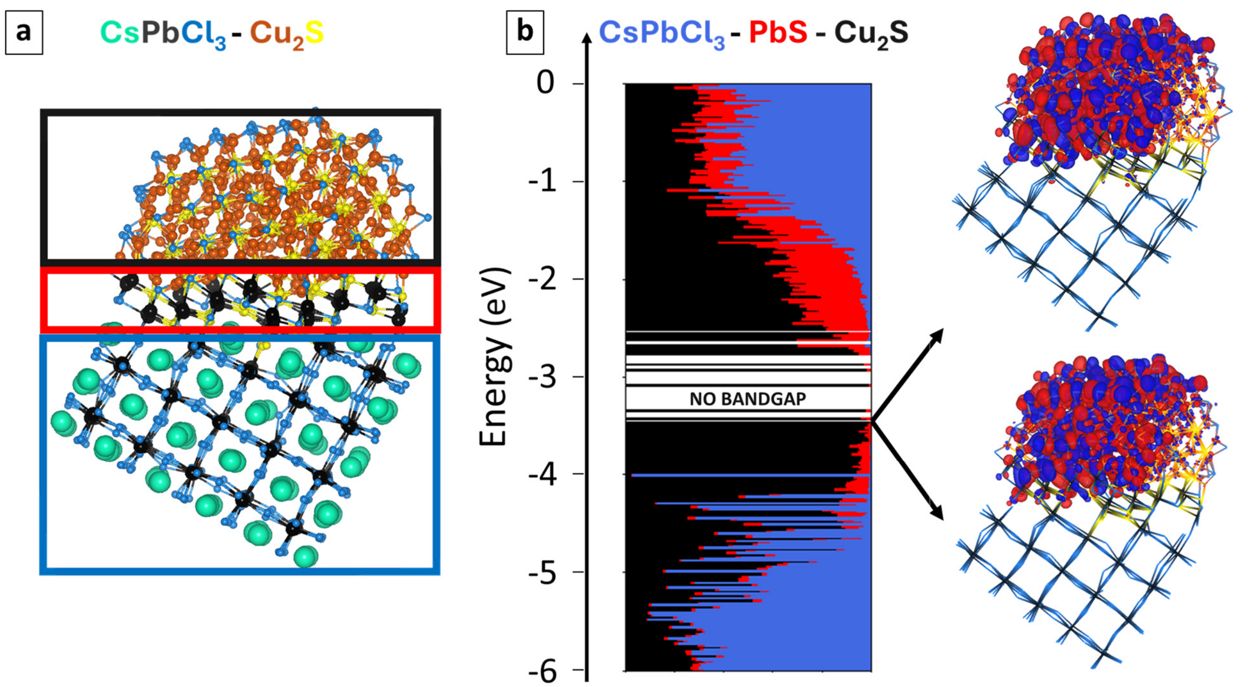

Figure S25. (a) Ball and sticks representation of the $CsPbCl_3$-Cu_2S NC heterostructure model optimized at the DFT/PBE level of theory: the presence of an intermediate PbS layer results in a smooth transition between the two domains as it allows the complete coordination of the ions of both $CsPbCl_3$ and Cu_2S domains at the interface. (b) Electronic structure of the model represented in (a) is computed at the DFT/PBE level of theory. The color code indicates the contribution of each domain to each molecular orbital. On the right, we plotted the frontier molecular orbitals. The absence of bandgap is most likely due to the presence of surface defects and to an underestimation of the band gap typical of some of the DFT exchange-correlation functionals, like the PBE employed here.
